# Structural determinants for activation of the Tau kinase CDK5 by the serotonin receptor 5-HT7R

**DOI:** 10.1186/s12964-024-01612-y

**Published:** 2024-04-19

**Authors:** Jana Ackmann, Alina Brüge, Lizaveta Gotina, Sungsu Lim, Kathrin Jahreis, Anna-Lena Vollbrecht, Yun Kyung Kim, Ae Nim Pae, Josephine Labus, Evgeni Ponimaskin

**Affiliations:** 1 Department of Cellular Neurophysiology, Institute for Neurophysiology, Hannover Medical School, Carl-Neuberg-Str. 1, 30625 Hannover, Germany; 2 Brain Science Institute, Korea Institute of Science and Technology (KIST), Seoul, Republic of Korea; 3 Division of Bio-Medical Science & Technology, KIST School, Korea University of Science and Technology (UST), Daejeon, Republic of Korea

**Keywords:** Serotonin receptor 7 (5-HT7R), Cyclin‐dependent kinase 5 (CDK5), Tau protein (Tau) and tauopathy, Site-directed mutagenesis, Computational modeling, Protein–protein complex, Interaction interface

## Abstract

**Background:**

Multiple neurodegenerative diseases are induced by the formation and deposition of protein aggregates. In particular, the microtubule-associated protein Tau leads to the development of so-called tauopathies characterized by the aggregation of hyperphosphorylated Tau within neurons. We recently showed that the constitutive activity of the serotonin receptor 7 (5-HT7R) is required for Tau hyperphosphorylation and aggregation through activation of the cyclin-dependent kinase 5 (CDK5). We also demonstrated physical interaction between 5-HT7R and CDK5 at the plasma membrane suggesting that the 5-HT7R/CDK5 complex is an integral part of the signaling network involved in Tau-mediated pathology.

**Methods:**

Using biochemical, microscopic, molecular biological, computational and AI-based approaches, we investigated structural requirements for the formation of 5-HT7R/CDK5 complex.

**Results:**

We demonstrated that 5-HT7R domains responsible for coupling to Gs proteins are not involved in receptor interaction with CDK5. We also created a structural model of the 5-HT7R/CDK5 complex and refined the interaction interface. The model predicted two conserved phenylalanine residues, F278 and F281, within the third intracellular loop of 5-HT7R to be potentially important for complex formation. While site-directed mutagenesis of these residues did not influence Gs protein-mediated receptor signaling, replacement of both phenylalanines by alanine residues significantly reduced 5-HT7R/CDK5 interaction and receptor-mediated CDK5 activation, leading to reduced Tau hyperphosphorylation and aggregation. Molecular dynamics simulations of 5-HT7R/CDK5 complex for wild-type and receptor mutants confirmed binding interface stability of the initial model.

**Conclusions:**

Our results provide a structural basis for the development of novel drugs targeting the 5-HT7R/CDK5 interaction interface for the selective treatment of Tau-related disorders, including frontotemporal dementia and Alzheimer’s disease.

**Supplementary Information:**

The online version contains supplementary material available at 10.1186/s12964-024-01612-y.

## Background

Multiple neurodegenerative disorders, including Alzheimer’s disease (AD), Parkinson’s disease, and frontotemporal dementia (FTD), are characterized by the formation of protein aggregates inside and outside of neurons, which results in progressive neuronal death and cognitive decline. In particular, aggregation of the microtubule-associated protein Tau leads to the development of so-called tauopathies, in which hyperphosphorylated and aggregated Tau protein accumulates within neurons [1]. The most prominent members of this class of diseases, which account for the majority of dementia cases worldwide, are AD and FTD. Tau aggregates are also found in several other neurodegenerative diseases, including Pick disease, progressive supranuclear palsy, corticobasal degeneration, and frontotemporal dementia with parkinsonism linked to chromosome 17 (FTDP-17) [2]. Under physiological conditions, Tau participates in the regulation of microtubule network stability and promotes tubulin polymerization, which influences cell morphology, axonal outgrowth, and axonal cargo transport [3]. In addition, Tau seems to play an important role for normal synapse function, by interacting with postsynaptic proteins, such as the PSD95-NMDA receptor complex, and F-actin [4, 5], and is critically involved in learning and memory processes [6, 7]. The functions of Tau are mainly regulated by its post-translational modification, particularly by phosphorylation at multiple sites [8]. Hyperphosphorylation of Tau leads to destabilization of the microtubule network, affecting axonal transport, proteasomal degradation pathways, as well as mitochondrial and synaptic function [9, 10]. More importantly, Tau hyperphosphorylation promotes its aggregation into neurotoxic Tau oligomers and the formation of neurofibrillary tangles, the neuropathologic hallmark of tauopathies [2, 3, 8].

Among the Tau kinases involved in pathological Tau hyperphosphorylation, the serine-threonine kinase cyclin-dependent kinase 5 (CDK5) has been implicated in AD pathogenesis [11]. CDK5 is activated by the binding to specific partners, p35 and p39, and their cleaved fragments, p25 and p29 [12]. The association of CDK5 with p25 is more stable and leads to aberrant hyperphosphorylation of substantial CDK5 substrates, including Tau [11]. In addition, CDK5 activity is regulated by its post-translational modifications, including serine phosphorylation at position 159 [13].

We have recently shown that the constitutive activity of the serotonin receptor 7 (5-HT7R) leads to activation of the CDK5. We also elucidated the underlying molecular machinery by demonstrating a physical interaction between 5-HT7R and CDK5 leading to receptor-dependent CDK5 activation, which induced aberrant Tau hyperphosphorylation, aggregation and neuronal death, finally resulting in cognitive decline [14]. In the present study, we investigated the structural requirements for the formation of 5-HT7R/CDK5 complex. Using computational prediction of the potential 5-HT7R/CDK5 interaction interface and molecular dynamics simulation in combination with site-directed mutagenesis, we identified two phenylalanine residues within the third intracellular loop of the 5-HT7R, F278 and F281, to be critically involved in 5-HT7R/CDK5 complex formation and CDK5 activation. More importantly, replacement of these phenylalanines by alanines significantly reduced receptor-mediated Tau hyperphosphorylation and aggregation without affecting Gs protein-mediated signaling.

## Methods

### Recombinant DNA procedures

All 5-HT7R constructs were labeled with different tags: N-terminal hemagglutinin (HA)-tag, C-terminal eYFP-tag or C-terminal mCherry-tag in a pcDNA3.1( +) vector. HA-tagged 5-HT7R ΔR395 was amplified from the wild-type (WT) construct using the indicated primers (Sigma) and inserted into the pcDNA3.1( +) vector after restriction digest with BamHI and XbaI enzymes. All other 5-HT7R mutant constructs were generated using an overlap-extension PCR protocol previously described [15]. Multiple mutations were introduced step-wise. Sequences of used primers are shown in Additional file 1. All derived constructs were verified by Sanger Sequencing (GATC-Biotech).

### Cell culture

The murine neuroblastoma cell line N1E-115 was cultured as previously described [14]. Transient transfection was performed using Lipofectamine 2000 reagent (Life Technologies) according to the manufacturer´s instructions. For transfection, we used plasmids encoding: eYFP, CDK5-eCFP, CDK5-mCherry, eGFP-Tau[R406W] as well as the above mentioned 5-HT7R WT and mutants under the control of a CMV promotor. For treatments, we applied 10 µM serotonin (Sigma), 50 µM 3-Isobutyl-1-methylxanthin (Tocris) and 5 µM forskolin (Sigma).

### Live-cell imaging

For analysis of subcellular protein localization, living cells were imaged using a Zeiss LSM780 confocal microscope (Carl Zeiss) equipped with a 40 × water immersion objective (C-Apochromat 40x/1.20 W Korr M27) with laser excitation of 440 nm for eCFP, 488 nm for eGFP and 514 nm for eYFP. Cell nuclei were visualized using 5 µg/ml Hoechst33342 (Invitrogen), incubated for 10 min, and 405 nm laser excitation. The subcellular distribution of CDK5 and 5-HT7R were analyzed using the ImageJ software. Maximum intensity projections of 10 µm thickness were generated. Intensity profiles of 40 µm length and 3 µm width were calculated and are displayed as relative values. Quantification of cells with Tau aggregates was performed as described previously [14]. Conditions to be compared were acquired with the same settings and processed in the same way using ImageJ software.

### cAMP measurements

For monitoring cellular cAMP responses, a FRET-based cAMP biosensor was applied [16]. Neuroblastoma cells were co-transfected with cAMP biosensor together with mRuby-5-HT7R WT or mutants. Live cell imaging over time was performed by exciting the fluorescent proteins with a 488 nm and a 561 nm laser as described previously [17]. Baseline of 3 min was captured prior to 5-HT (Tocris) perfusion. Amplitude and decay time τ for each cell was calculated using a single exponential fit with a polynomial offset described previously [17].

### Immunoblotting

For analysis of 5-HT7R protein expression, transfected N1E-115 cells were lysed in RIPA buffer (1% Triton X-100, 1% sodium deoxycholate, 0.1% SDS, 150 mM NaCl, 20 mM Tris/HCl (pH 7.4), 10 mM EDTA, 10 mM iodoacetamide (pH 7.4), 1% CLAP and PMSF). For the analysis of Tau and CDK5 phosphorylation, cells were lysed in lysis buffer (5 mM EDTA, 20 mM HEPES, 100 mM NaCl, 100 mM NaF, 1 mM Na_3_VO_4_, 1% Triton X-100). Samples were centrifuged at 18,000 g for 15 min at 4 °C, and the supernatant was mixed with 2 × Thorner buffer (0.4 mg/mL bromophenol blue, 0.1 mM EDTA, 5% SDS, 40 mM Tris/HCl pH 6.8, 8 M urea, 1% ß-mercaptoethanol) or with 6 × SDS loading buffer (30% glycerol, 10% SDS, 0.35 M Tris pH 6,9, 5% ß-mercaptoethanol). Proteins were denaturated at 56 °C for 15 min and at 95 °C for 5 min, respectively, and further analyzed by SDS-PAGE and immunoblotting. For the Western blot analysis, equal amounts of protein were separated on SDS–polyacrylamide gels. Proteins were then transferred onto nitrocellulose membranes and probed with the following antibodies: HA (peroxidase-conjugated, 1:500, Roche), mCherry (1:1,000, SICGEN), GFP (HRP-conjugated, 1:5,000, Biozol), total Tau 5A6 (1:100, DSHB Hybridoma), pThr181 Tau AT270 (1:1,000, Thermo Fisher Scientific), total CDK5 (1:500. MyBioSource), pSer159 CDK5 (1:250, Santa Cruz). GAPDH antibody (1:10,000, Gene Tex) was used as loading control for all experiments. Western blot signals were densitometrically quantified using a custom-written MatLab script and normalized by the sum of the replicates.

### Co-Immunoprecipitation assay

Co-Immunoprecipitation was performed according to a modified protocol previously established [18]. In brief, N1E-115 cells were co-transfected with mCherry-tagged 5-HT7R and eCFP-tagged CDK5 constructs and lysed in RIPA buffer. Lysates were incubated overnight either with 3 µg mCherry-antibody (goat, SICGEN) or IgG (goat, Sigma) as a control. Precipitated proteins were incubated with proteinA sepharose (Sigma) for two hours followed by extensive washing. Proteins were eluted in Thorner buffer for 20 min at 37 °C and analyzed by SDS-PAGE and immunoblotting.

### ColabFold model generation

A total of 3 protein complexes were modeled: *Homo sapiens* 5-HT7R/CDK5 (*h*5-HT7R/CDK5), *Mus Musculus* 5-HT7R/CDK5 (*m*5-HT7R/CDK5), and 5-HT7R/Gɑs (Gs alpha subunit coupled with 5-HT7R). The initial complex structures were predicted using the publically available platform ColabFold v1.5.2 [19], which replaces the homology detection and MSA pairing of AlphaFold2 [20, 21] with MMseqs2 [22–24]. The query protein sequences were taken from corresponding Uniprot entries with some truncation as follows: *h*5-HT7R/CDK5—P34969 (aa69-413) & Q00535 (aa1-292); *m*5-HT7R/CDK5—P32304 (aa72-416) & P49615(aa1-292), 5-HT7R/Gɑs—P32304 (aa71-405) & P63092 (aa42-394). Parameters of the model generation were as follows: msa_mode – mmseqs2_uniref_env, pair_mode – unpaired-paired, model type—alphafold2_multimer_v2, number of recycles – 48, template mode—pdb70 [25] and num_relax – 5. This combination of settings produced 5 energy-relaxed models, whose structure was based on a maximum of 20 top-ranking target templates from a clustered version of the PDB database. For 5-HT7R/Gɑs model the top ranking complex was chosen, whereas 5-HT7R/CDK5 models were clustered based on protein backbone similarity into 3 groups (Additional file 2). In each group, the protein with highest protein–protein interaction area was chosen as a representative structure. The representative structures were superimposed onto the CDK5/p25 crystal structure 1H4L in such a way to maximize overlap of CDK5 units, after which overlapping areas of p25 and 5-HT7R were analyzed.

### Model energy minimization and side chain optimization

The chosen ColabFold models were refined in Discovery Studio 2021 (BIOVIA, Dassault Systèmes, 2020). Refinement consisted of protein–protein interface (PPI) energy minimization, loop refinement (only for models which include CDK5), and side-chain refinement. The PPI interface was detected and analyzed by the DS Protein Interface tool. The protein–protein contact area was employed for PPI detection with an interface distance cutoff of 6 Å. Afterwards, the detected PPI area was enlarged to additionally include residues within 6 Å radius of the discovered contact area. The energy minimization and side chain refinement were applied to the enlarged area. In the minimization stage, the Smart Minimization algorithm was employed, which combines 1000 steps of steepest descent with an RMS gradient tolerance of 3, followed by Conjugate Gradient minimization. Max steps were set to 3000 and the RMS gradient to 0.03. Loop refinement (DS CHARMm-based improved LOOPER algorithm [26]) was applied to the CDK5 activation loop residues 148–161. Lastly, side chain refinement was performed using the ChiRotor algorithm [27] implemented in DS with CHARMm forcefield. A total of 133, 130, and 171 residues were refined in the *h*5-HT7R/CDK5, *m*5-HT7R/CDK5, and 5-HT7R/Gs models, respectively. The model refinement was considered successful as the PPI area increased, and any initially present unfavorable interactions were eliminated (data not shown).

### Molecular dynamics (MD) simulation

The 5-HT7R ICL3 refinement and protein complex stability were approached through molecular dynamics simulations. The protein preparation, positioning, and solvation with an explicit POPC lipid membrane, as well as generation of system topology, and parameters for conducting the equilibration and production phases were all done using the CHARMM-GUI generator Membrane Builder [28–30]. The MD system composition details (size, lipid count, number of ions, and water molecules) are provided in Additional file 3. Simulations were performed using the GROMACS 2016.4 package [31, 32] under the CHARMM36m forcefield [33, 34]. PME (particle mesh Ewald) was used to evaluate long-range electrostatics with a cutoff of 1.2 nm. The systems were minimized using the steepest descent algorithm with a maximum of 5000 steps until convergence to a maximum force of 1000 kJ∙mol^−1^∙nm^−1^. Equilibration at 310 K and 1 bar was performed in 6 steps (250 ps each) using the Berendsen thermostat, Berendson semi-isotropic pressure coupling (coupling constant 5.0 and compressibility 4.5∙10^−5^ bar^−1^), and applying LINCS h-bond constraints. During the 6-step equilibration, harmonic force restraints were gradually released from 4000 to 0 kJ∙mol^−1^∙nm^−2^. Production was run for 400 ns using a Nose’-Hoover thermostat and a Parrinello–Rahman barostat with semi-isotropic coupling (coupling constant 5.0 and compressibility 4.5∙10^−5^ bar^−^1). 3D periodic boundary conditions were applied.

### Mutation energy (binding) calculation

For each 5-HT7R/CDK5 complex model, 10 screenshots were taken from the last 10 ns of the MD simulation and imported into Discovery Studio software (a total of 20 screenshots). For each screenshot, all 5-HT7R amino acids involved in the protein complex interface were selected and submitted to the DS Calculate Mutation Energy tool [35]. As written in the software description, this tool calculates the energy effect of a mutation on the binding affinity (mutation energy, ΔΔG_mut_) as the difference between the binding free energy in the mutated structure and WT protein. The CHARMm Polar H forcefield and Generalized Born implicit solvent model were used to calculate the energy terms, and other tool parameters were kept default. Single point mutations to alanine were calculated. High, positive mutation energy values above 0.5 kcal/mol indicate a destabilizing effect of the mutation. The resulting average value of mutation energy was reported as representative of the last 10 ns of the performed MD simulation for each 5-HT7R amino acid of interest.

### Statistical analysis

Statistical analysis was performed using GraphPad Prism version 7 (La Jolla, CA, USA). Data are presented as mean and standard deviation (SD). Unless stated otherwise, group-wise datasets were tested for significant differences using one-way analysis of variance (ANOVA) followed by Dunnett´s multiple comparison test and pairwise datasets by unpaired *t*-test after testing Gaussian distribution by Shapiro–Wilk normality test. The following *p* values were considered to be statistically significant: *: *p* < 0.05, **: *p* < 0.01, ***: *p* < 0.001.

## Results

### 5-HT7R recruits CDK5 to the plasma membrane, and 5-HT7R/CDK5 complex formation does not require the Gs protein

**Fig. 1:**
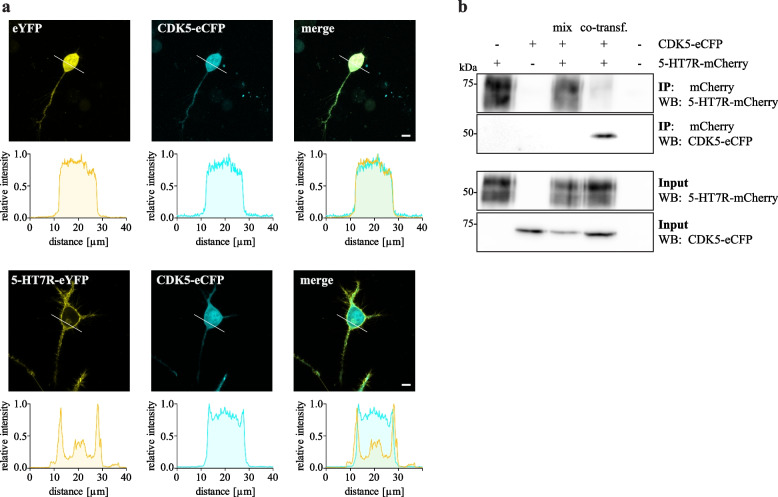
5-HT7R and CDK5 interact and are co-localized at the plasma membrane. **A** Representative confocal images showing N1E-115 cells co-expressing either CDK5-eCFP and eYFP (control, upper panel) or CDK5-eCFP and 5-HT7R-eYFP (lower panel). Corresponding intensity profiles are shown below. Scale bar: 10 μm. See also Additional file 4. **B** N1E-115 cells were co-transfected with eCFP-tagged CDK5 and mCherry-tagged 5-HT7R, followed by immunoprecipitation (IP) with anti-mCherry antibody and Western blot with anti-CFP antibody. As a control, mixed lysates from the single transfected cells (mix) were applied to the IP

We have recently shown that the constitutive 5-HT7R activity is required for Tau hyperphosphorylation and aggregation through the CDK5-dependent mechanism [14, 36]. Since CDK5 localization and activity is known to be modulated by interaction with its regulatory proteins [37, 38], we analyzed whether the 5-HT7R might influence subcellular distribution of CDK5. To this end, neuroblastoma N1E-115 cells were co-transfected with eCFP-labeled CDK5 together with either eYFP-labeled 5-HT7R or eYFP (control) followed by confocal microscopy analysis (Fig. 1A). In absence of the 5-HT7R, CDK5 was diffusely distributed in the cell with only a small portion resided at the membrane. In contrast, expression of the 5-HT7R leads to a prominent shift of CDK5 to the plasma membrane, where it was co-localized with the 5-HT7R (Fig. 1A, Additional file 4).

To validate a complex formation between 5-HT7R and CDK5, we performed co-immunoprecipitation (co-IP) experiments in N1E-115 cells co-expressing mCherry-tagged 5-HT7R and eCFP-tagged CDK5. Figure 1B shows that after immunoprecipitation with an antibody against the mCherry-tag, the eCFP-tagged CDK5 were identified only in samples derived from cells co-expressing both mCherry- and eCFP-tagged proteins. To exclude effects of artificial protein aggregation, cells expressing only one type of protein (i.e. either mCherry-5-HT7R or eCFP-tagged CDK5) were mixed prior to lysis and analyzed in parallel (“mix” samples). As shown in Fig. 1B, both 5-HT7R and CDK5 can be detected by the corresponding antibody (visible in “input” fraction), but no co-IP was observed. This verifies the specificity of 5-HT7R/CDK5 interaction and is in line with our previous observations [14].

The 5-HT7R mediates its cellular responses by the activation of two different heterotrimeric G proteins, Gs and G12 [39, 40]. While the involvement of the G12 protein in 5-HT7R-induced CDK5-mediated Tau pathology was already excluded [14], it has not been investigated whether Gs protein participates in the 5-HT7R/CDK5 complex formation. Therefore, we next performed co-IP experiments after selective knockdown of the Gαs subunit by specific short hairpin RNAs (shRNA; Additional file 5) [41]. Quantitative analysis of co-IP experiments revealed that silencing of Gαs does not result in any significant differences in the amount of co-precipitated 5-HT7R after CDK5-mCherry pull-down (Additional file 5), suggesting that Gs protein is not involved in 5-HT7R/CDK5 complex formation.

### 5-HT7R domains responsible for coupling with Gs protein are not involved in receptor interaction with CDK5

It has been demonstrated that the 5-HT7R can pre-associate with Gs protein [42] and that its intracellular loop 3 (ICL3) as well as its C-tail are mainly responsible for such pre-coupling [43]. In particular, the charged amino acids E325 and K327 located within the ICL3 have been shown to play a critical role in this interaction [44].

**Fig. 2:**
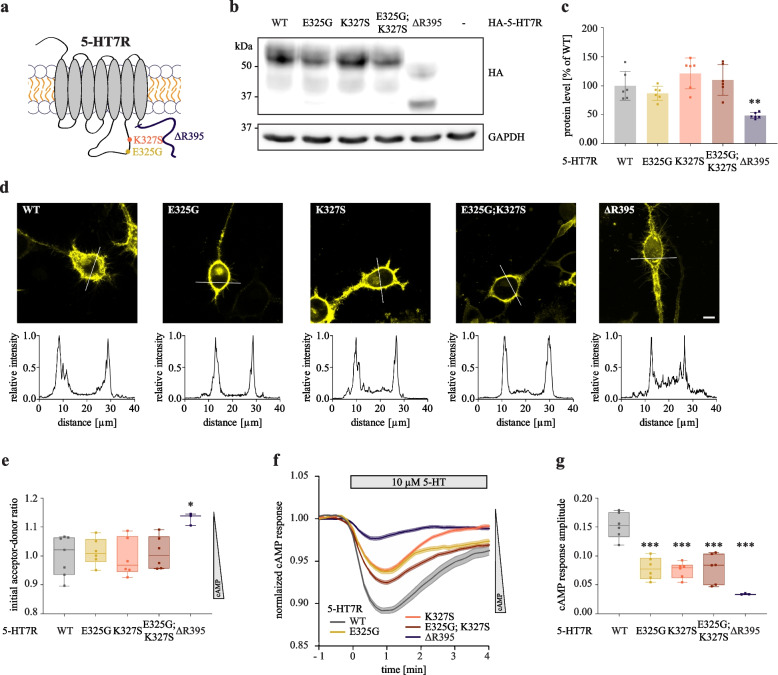
Expression and functional characterization of 5-HT7R mutants with impaired Gs coupling. **A** Scheme of receptor mutants. Substitution mutants E325G, K327S and E325G;K327S possess single or double exchanged amino acids in the ICL3, whereas ΔR395 lacks the carboxy-terminal domain. **B**, **C** Expression of HA-tagged 5-HT7R WT or mutants in N1E-115 cells. Representative Western blot (**B**) and quantification (**C**). The 5-HT7R levels were normalized to GAPDH and are shown as mean ± SD (N = 6, one-way ANOVA, Dunnett’s multiple comparisons, *** *p* < 0.01). **D** Representative confocal images showing expression of eYFP-tagged 5-HT7R WT or mutants. Corresponding intensity profiles are shown below. Scale bar: 10 μm. See also Additional file 6. **E** N1E-115 cells were transfected with cAMP fluorescence resonance energy transfer-based biosensor CEPAC and indicated constructs of eYFP-tagged 5-HT7R. Basal cAMP levels are shown. Signals are normalized to cells transfected with 5-HT7R WT (3 ≤ N ≤ 7, one-way ANOVA, Dunnett’s multiple comparison, * *p* < 0.05). **F** Representative traces showing cAMP response at the single cell level after stimulation with 10 µM of serotonin (5-HT). **G** Graphs show cAMP response amplitude relative to pretreatment (3 ≤ N ≤ 7, one-way ANOVA, Dunnett’s multiple comparisons, *** *p* < 0.001)

To verify whether the same receptor domains can also be involved in the interaction with CDK5, we introduced the single mutations E325G and K327S in the ICL3 of 5-HT7R (Fig. 2A). We also established a mutant receptor combining both substitutions (E325G;K327S). In addition, we generated a 5-HT7R deletion mutant lacking the C-terminal domain starting from arginine in position 395 (ΔR395, Fig. 2A). As shown in Fig. 2B and C, E325G, K327S and E325G;K327S mutants showed similar expression levels as the WT receptor. In contrast, deletion of the C-terminal domain significantly reduced expression levels of truncated receptor (Fig. 2B and C; WT: 1.00 ± 0.25 vs. ΔR395: 0.48 ± 0.05). Of note, none of the mutations influenced the localization of the receptor at the plasma membrane (Fig. 2D, Additional file 6). We next studied the ability of these mutants to modulate cAMP production using the FRET-based cAMP biosensor CEPAC [16]. In this sensor, a low cAMP concentration correlates with a high FRET signal between mCerulean (FRET donor) and Citrine (FRET acceptor), while an increase in cAMP concentration results in decreased FRET efficiency, which is depicted as increased acceptor-to-donor ratio (Additional file 7A and B). Since 5-HT7R possesses a high constitutive activity towards the Gs protein-dependent signaling, we first analyzed the acceptor-to-donor ratios of CEPAC biosensor in cells expressing WT or mutants of 5-HT7R under basal conditions. While the substitution mutants E325G, K327S and E325G;K327S showed no differences compared to WT, ΔR395 demonstrated a higher initial acceptor-to-donor ratio, which corresponds to lower cAMP levels (Fig. 2E; WT: 1.00 ± 0.07 vs. ΔR395: 1.13 ± 0.02). This might result from lower 5-HT7R ΔR395 expression levels, but could also be a consequence of reduced pre-association of this mutant with Gs proteins. Next, we compared serotonin-induced cAMP response by quantification of the response amplitude as well as the activation decay time [17]. All mutants showed a significantly reduced cAMP response amplitudes with the most prominent effect obtained in the ΔR395 mutant (Fig. 2F and G; WT: 0.152 ± 0.022 vs. E325G: 0.078 ± 0.019, K327S: 0.075 ± 0.013, E325G;K327S: 0.079 ± 0.025 vs. ΔR395: 0.034 ± 0.001), while response kinetics was not affected (Additional file 7C).

**Fig. 3:**
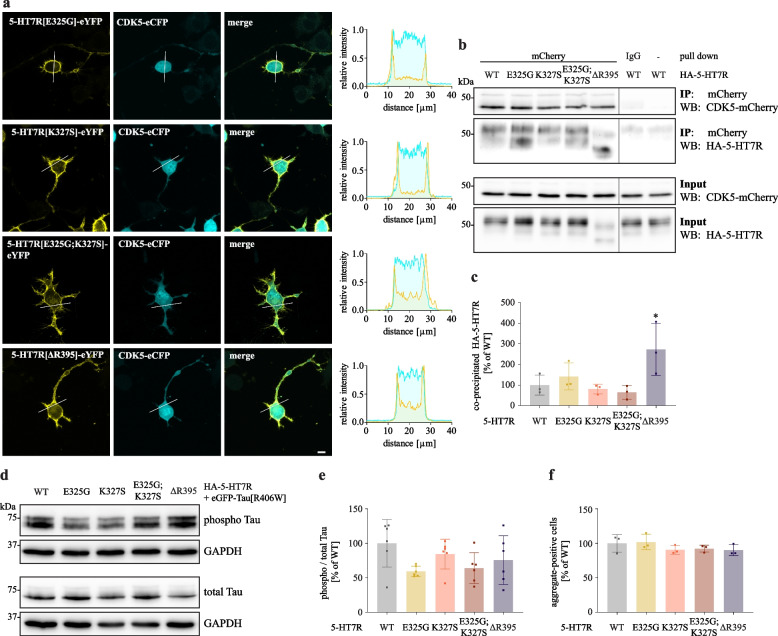
5-HT7R domains responsible for coupling with Gs protein are not involved in interaction and activation of CDK5. **A** Representative confocal images of N1E-115 cells co-expressing either CDK5-eCFP and eYFP-5-HT7R mutants E325G, K327S, E325G;K327S or ΔR395. Scale bar: 10 μm. Corresponding intensity profiles are shown on the right. See also Additional file 8. **B**, **C** N1E-115 cells were co-transfected with mCherry-tagged CDK5 and HA-tagged 5-HT7R constructs as indicated, followed by IP with anti-mCherry antibody and Western blot with anti-HA antibody. Quantification of the co-IP experiments is shown below. The ratio of co-precipitated receptor was calculated, normalized to the WT sample and is presented as mean ± SD (N = 3, Kruskal–Wallis test, Dunn’s multiple comparisons, no statistical significance to WT). **D**, **E** N1E-115 cells were transfected with eGFP-Tau[R406W] mutant, together with the indicated HA-tagged 5-HT7R constructs. Phospho-Tau and total Tau levels were detected with AT270 and 5A6 antibodies, respectively. Resulting ratios (**E**) were normalized to GAPDH expression and are shown as normalized mean ± SD (N = 4, Kruskal–Wallis test, Dunn’s multiple comparisons, no statistical significance to WT). **F** The number of Tau aggregate-positive cells was counted in a confined area and is presented as a fraction of the total number of transfected cells. Data is presented as normalized mean ± SD (N = 3, n ≥ 353, Kruskal–Wallis test, Dunn’s multiple comparisons, no statistical significance to WT)

In addition, we analyzed co-localization of eYFP-tagged 5-HT7R mutants with CDK5-eCFP after co-expression in neuroblastoma cells (Fig. 3A). Similar to the results obtained for the 5-HT7R WT, CDK5 was co-localized with all 5-HT7R mutants at the plasma membrane (Fig. 3A, Additional file 8) suggesting that Gs protein coupling is not required for the 5-HT7R/CDK5 interaction. This was further confirmed by the co-IP experiments, in which we did not observe any statistical differences between WT and mutants (Fig. 3B and C). These findings suggest that the receptor domains important for Gs protein coupling are not involved in the interaction of 5-HT7R with CDK5.

Next, we investigated whether mutations of residues involved in receptor-Gs protein coupling might influence 5-HT7R-mediated effects on Tau pathology. To this end, we overexpressed the eGFP-tagged human Tau[R406W] mutant associated with familiar cases of FTD [45–47] either with 5-HT7R WT or with abovementioned 5-HT7R mutants. In line with our previous observations, co-expression of Tau[R406W] mutant with 5-HT7R WT results in significantly increased Tau phosphorylation at the CDK5 target site Thr181. Noteworthy, neither substitution mutations in the ICL3 nor depletion of the C-terminus influence Tau phosphorylation levels (Fig. 3D and 3E).

Finally, we investigated the impact of the introduced mutations on 5-HT7R-induced Tau aggregation. We have recently shown that 5-HT7R expression results in the formation of highly bundled hyperphosphorylated Tau structures, which are dissociated from microtubules and resemble Tau aggregates in vitro (Fig. 3F) [14]. Neuroblastoma cells expressing eGFP-Tau[R406W] along with WT and mutant receptors were analyzed by confocal microscopy, and the fraction of Tau aggregate-containing cells (Additional file 7D) was calculated. In the presence of the 5-HT7R WT, about 41.6% ± 0.4% of the transfected cells were positive for Tau aggregates. Similar results were also obtained after expression of the E325G, K327S, E325G;K327S and ΔR395 mutants.

Taken together, our experiments revealed that receptor domains involved in Gs coupling and Gs protein-dependent signaling do not affect 5-HT7R/CDK5 interaction or receptor-mediated Tau hyperphosphorylation and aggregation.

### Computational prediction of the 5-HT7R/CDK5 interaction interface

Having excluded the involvement of receptor domains responsible for Gs protein coupling (i.e., E325, K327, and C-terminus) in the 5-HT7R/CDK5 interaction and subsequent Tau pathology, we sought to predict the 5-HT7R/CDK5 interaction interface using molecular modeling approaches. Considering the small surface area of the ICL1 and ICL2, the ICL3 of the 5-HT7R appears most likely to be involved in the interaction interface of the 5-HT7R/CDK5 complex. Despite the availability of experimental structures for 5-HT7R/Gs and CDK5/p25 complexes (PDB ID: 7XTC [48] and 1H4L [49], respectively) direct protein–protein docking of CDK5 to 5-HT7R is complicated by the absence of a resolved structure for the ICL3, as well as any homologous fragments to model it. The structural prediction algorithm AlphaFold-multimer [50] has shown promising results in predicting the structures of protein complexes [20, 51–53], greatly surpassing the accuracy of protein docking [54]. Therefore, we employed the freely available platform ColabFold [19], which combines the AlphaFold2 algorithm with MMseqs2 sequence alignment.

We first verified the applicability of ColabFold by predicting the structure of a 5-HT7R/Gs complex (Additional file 9C). The highest ranking model had a pTM score equal 0.80 and interface score iPTM 0.75, which was within the threshold corresponding to a confident model [54]. Areas of low confidence were obtained within the 5-HT7R ICL3 and C-terminal domain as well as the Gαs alpha helix. The protein complex backbone RMSD of the highest ranking model compared to the experimental structure was 2.767 Å (Additional file 10) with interface RMSD—2.121 Å, therefore classifying it under CAPRI acceptable model quality for protein–protein complexes [55]. Our model revealed an interaction of K327 with Gαs residues E392 and L394, which is in line with the experimentally determined structure [48]. In addition, glutamic acid E325 (absent in the experimental structure) participated in stabilizing the α-helical structure of TM5 and TM6, specifically by interacting with 5-HT7R residue R270. Interestingly, the high similarity of the model to the experimental structure was obtained from a diverse multi-template approach and did not include 7XTC as a template (Additional file 11).

We then generated five initial models for protein sequences of *Homo sapiens* 5-HT7R/CDK5 (*h*5-HT7R/CDK5) and *Mus Musculus* 5-HT7R/CDK5 (*m*5-HTR7/CDK5) complexes (Additional file 9A and B). These models had at best pTM scores equal 0.7 and iPTM equal 0.58 for *m*5-HT7R/CDK5 and pTM—0.66 and iPTM—0.57 for *h*5-HT7R/CDK5. Finally, we performed energy minimization and refinement using LOOPER and side chain refinement to optimize the residue interactions. Similar to the 5-HT7R/Gs complex, the individual structures of the 5-HT7R and CDK5 subunits in the complexes were highly similar to those determined experimentally with backbone RMSD at 1.99 Å (Additional file 10).

**Fig. 4:**
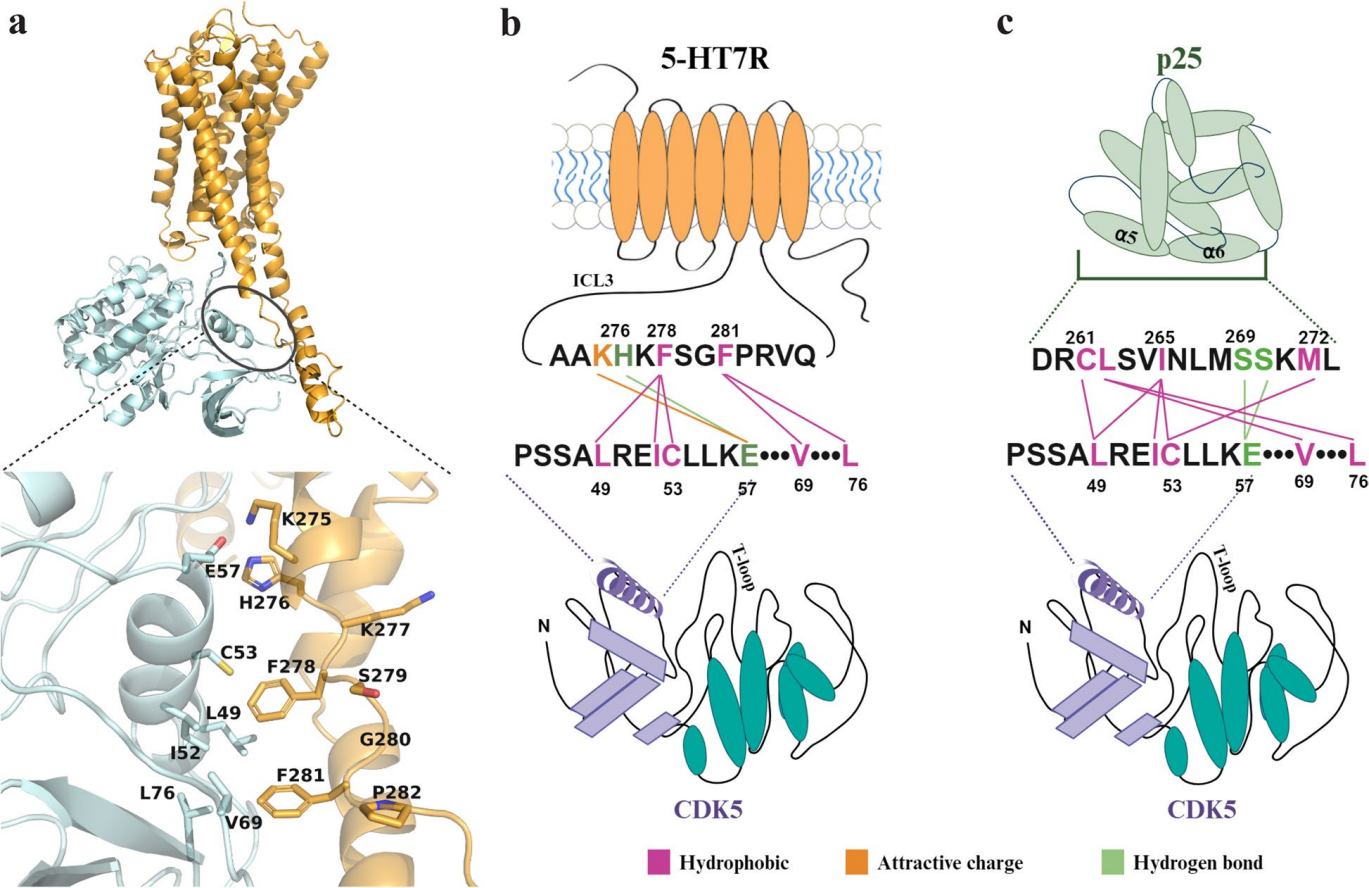
ColabFold 5-HT7R/CDK5 interaction interface prediction and comparison to CDK5/p25 complex. **A** General view of energy minimized ColabFold model for *m*5-HT7R/CDK5 protein complex. Enlarged view shows of contacts between ICL3 and CDK5 αC-helix. 5-HT7R is shown in orange and CDK5 in light cyan color. **B** Scheme depicts amino acid residues within *m*5-HT7R ICL3 and CDK5 αC-helix proposed to be involved in *m*5-HT7R/CDK5 interaction interface. **C**. Scheme depicts amino acid residues within α5 and α6 p25 domains and CDK5 αC-helix involved in formation of interaction interface (based on X-ray crystal structure 1H4L [53]. The α-helixes are shown as ovals and β-sheets as parallelograms. The CDK5 PSSALRE region is highlighted as a violet helix. Images in (**B**) and (**C**) were created using BioRender

Based on the complex architecture, all models were divided into 3 groups. To determine the more eligible complex, we compared representative complexes of each group with the known complex of CDK5 and its activator p25. In the group one type models, the structural elements of 5-HT7R demonstrated the best structural overlay with the binding interface of p25 (Additional file 2). Both the *m*5-HT7R/CDK5 and* h*5-HT7R/CDK5 models predicted that the binding interface between 5-HT7R and CDK5 largely consists of the residues within the ICL3 (Fig. 4A). According to this model, 5-HT7R residues 276–282 and 311–316 are in close contact with the CDK5 ɑC-helix (PSSALRE) and N-lobe. Compared to the experimentally solved CDK5/p25 complex, these domains structurally corresponded to ɑ5-ɑ6 helixes of p25. The tightest interaction with the ɑC-helix hydrophobic surface of CDK5 was exhibited by 5-HT7R residues F278 and F281 (Fig. 4B). Their interactions corresponded to p25 residues L262 and I265 (Fig. 4C). In addition, 5-HT7R residues K275 and H276 were within H-bonding distance of CDK5 residue E57, which also belongs to the interaction interface within the CDK5/p25 complex. However, the hydrophobic contacts of the phenylalanine residues covered the largest portion of the protein–protein interaction surface, in sum being more sufficient that a single H-bond.

### Mutagenesis of F278 and F281 does not affect receptor localization and Gs protein-dependent signaling but impairs interaction with CDK5

**Fig. 5:**
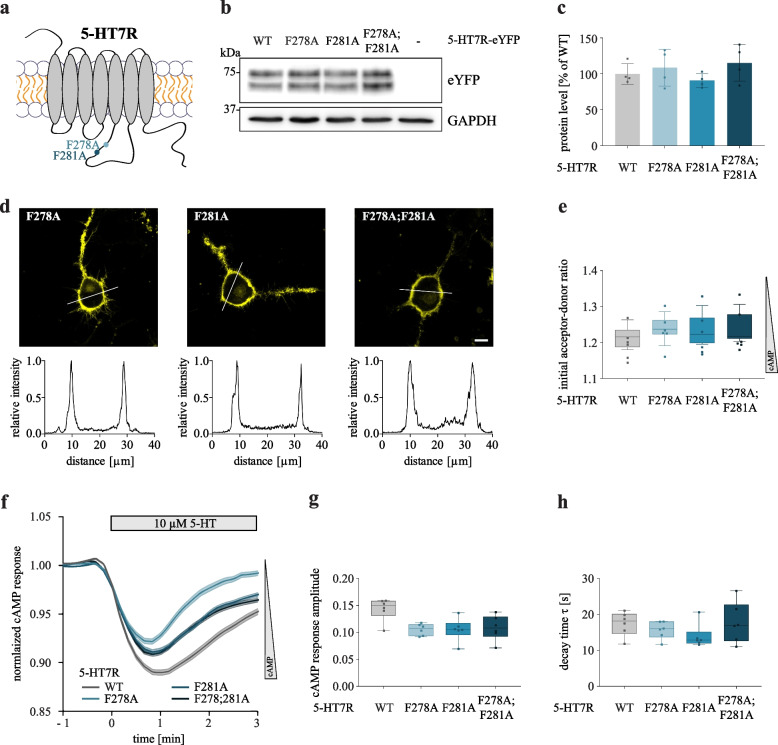
Characterization of 5-HT7R phenylalanine mutants. **A**. Scheme of 5-HT7R phenylalanine mutants. **B**, **C** Expression of HA-tagged 5-HT7R WT or indicated mutants in N1E-115 cells. Representative Western blot (**B**) and quantification (**C**) are shown. Data are presented as mean ± SD (N = 4, one-way ANOVA, Dunnett’s multiple comparisons. **D** Representative confocal images showing expression of eYFP-tagged 5-HT7R mutants. Corresponding intensity profiles are shown below. Scale bar: 10 μm. See also Additional file 12. **E** N1E-115 cells were transfected with cAMP FRET-based biosensor CEPAC and indicated eYFP-tagged 5-HT7R constructs. Basal cAMP levels are shown. Signals are normalized to cells transfected with 5-HT7R WT (N = 6, one-way ANOVA, Dunnett’s multiple comparisons, no statistical significance to WT). **F** Representative traces showing cAMP response at the single cell level after stimulation with 10 µM of 5-HT. **G**, **H** Graphs show response amplitude (**G**) and activation time constant (**H**) of cAMP relative to pretreatment (*N* = 6, one-way ANOVA, Dunnett’s multiple comparisons, no statistical significance to WT)

Modeling results of the 5-HT7R/CDK5 complex predict that the ICL3 is in close proximity to the CDK5 ɑC-helix and that this contact is largely facilitated by hydrophobic interactions of F278 and F281. To validate the structural model, we generated substitution mutations of residues F278 and F281 (either separately or in combination). The phenylalanine residues were replaced with alanine, which largely preserves the carbon backbone structure (Fig. 5A).

Expression levels and subcellular localization of the F278A, F281A, and F278A; F281A mutants were comparable to those obtained for the WT 5-HT7R (Fig. 5B-D, Additional file 12). We also verified Gs protein-mediated signaling of the mutated receptors using the FRET-based cAMP biosensor CEPAC. For all mutants, we found no differences in the initial acceptor-to-donor ratios compared to the neuroblastoma cells expressing WT 5-HT7R (Fig. 5E). In addition, the 5-HT-evoked cAMP responses were not affected (Fig. 5F-H), indicating that F278 and F281 are not involved in the Gs protein-mediated 5-HT7R signaling.

**Fig. 6:**
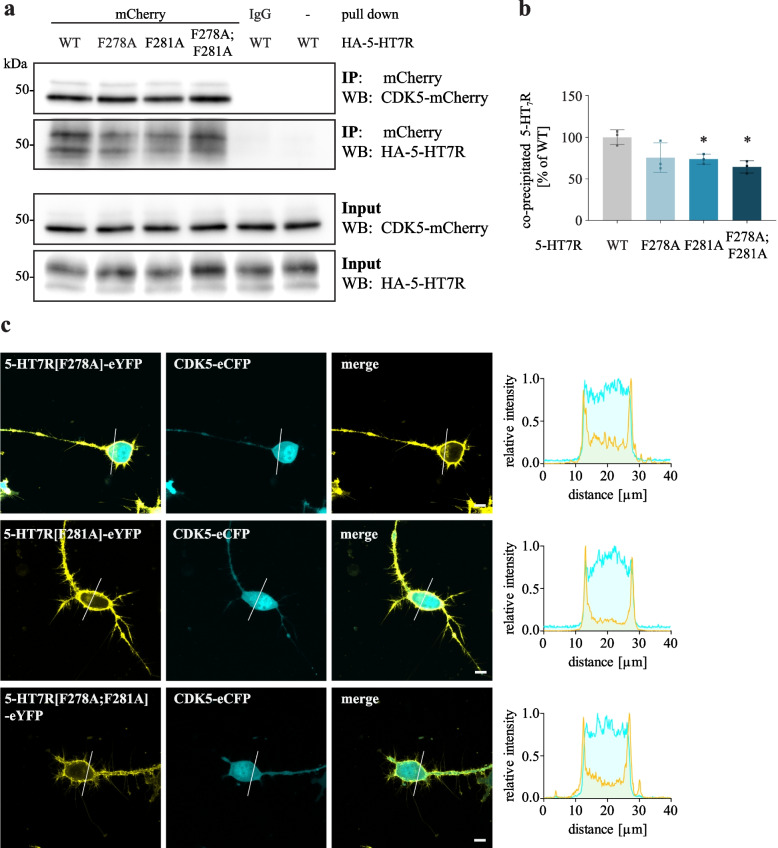
Mutations of F278A and F281A impairs interaction of 5-HT7R with CDK5. **A**, **B** N1E-115 cells were co-transfected with mCherry-tagged CDK5 and HA-tagged 5-HT7R constructs as indicated, followed by IP with anti-mCherry antibody and Western blot with anti-HA antibody. Quantification of the co-IP experiments is shown on the right. Ratios of co-precipitated receptors was calculated, normalized to the WT sample and are presented as mean ± SD (N = 3, one-way ANOVA, Dunnett’s multiple comparisons, * *p* < 0.05). **C** Representative confocal images of N1E-115 cells co-expressing either CDK5-eCFP and eYFP-5-HT7R mutants F278A, F281A or F278A; F281A. Scale bar: 10 μm. Corresponding intensity profiles are shown on the right. See also Additional file 13

To test whether the phenylalanine residues are involved in the interaction of 5-HT7R with CDK5, we performed co-IP experiments in neuroblastoma cells co-expressing mCherry-CDK5 with either HA-tagged 5-HT7R WT or mutants (Fig. 6). As shown in Fig. 6A and B, we obtained a significant decrease in the interaction of 5-HT7R with CDK5 for F281A and F278A;F281A mutants compared to WT (WT: 100% ± 8.7% vs. F281A: 73.8% ± 6.0%, F278;F281A: 64.5% ± 7.4%). In line with this, we found that upon co-expression of the 5-HT7R mutants with CDK5, the latter was mainly localized in the cytoplasm. In particular, in cells expressing the F278A;F281A mutant, only a negligible population of CDK5 was recruited to the plasma membrane (Fig. 6C, Additional file 13). These results suggest that the phenylalanine residues within the ICL3 contribute to 5-HT7R interaction with CDK5.

### Impaired 5-HT7R/CDK5 interaction diminishes receptor-mediated Tau phosphorylation and aggregation

**Fig. 7:**
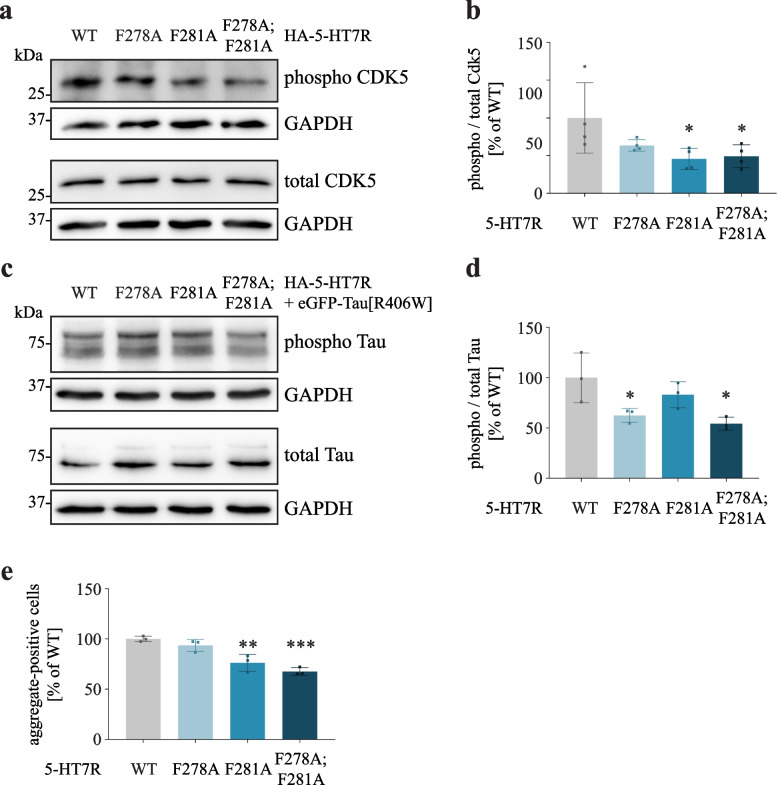
Mutations of F278A and F281A ameliorates 5-HT7R-induced Tau pathology. **A**, **B** Phosphorylation levels of endogenous CDK5 in N1E-115 expressing HA-tagged 5-HT7R WT or indicated phenylalanine mutants. Representative Western blot (**A**) and quantification (**B**). Data is represented as mean ± SD (N = 4, one-way ANOVA, Dunnett’s multiple comparisons, * *p* < 0.05). **C**, **D**. N1E-115 cells were transfected with eGFP-Tau[R406W] mutant, together with indicated HA-tagged 5-HT7R constructs. Phospho-Tau and total Tau levels were detected with AT270 and 5A6 antibodies, respectively. Resulting ratios (**D**) were normalized to GAPDH expression and are shown as normalized mean ± SD (N = 3, one-way ANOVA, Dunnett’s multiple comparisons, * *p* < 0.05). **E** The number of Tau aggregate-positive cells was counted in a confined area and is presented as a fraction of the total number of transfected cells. Data is presented as normalized mean ± SD (N = 3, n ≥ 353, Dunnett’s multiple comparisons, ** *p* < 0.01, *** *p* < 0.001)

Having demonstrated the involvement of 5-HT7R residues F278 and F281 in the interaction with CDK5, we investigated whether their substitutions might affect CDK5 activation. Phosphorylation of CDK5 at residue S159 is known to increase its activity [56–58]. Therefore, we analyzed 5-HT7R-mediated phosphorylation of S159 as a read-out for CDK5 activation. As shown in Fig. 7A and B, phosphorylation of CDK5 at S159 was decreased in all 5-HT7R phenylalanine mutants reaching significant levels for the F281A and F278A;F281A mutants (WT: 100% ± 46.8% vs. F281A: 45.7% ± 14.1%, F278A;F281A: 49.2% ± 15.2%). This demonstrates that reduced interaction of 5-HT7R with CDK5 results in impaired CDK5 activation.

To investigate possible consequences of reduced CDK5 activation on Tau pathology, we co-transfected neuroblastoma cells with eGFP-tagged Tau[R406W] mutant along with either WT 5-HT7R or phenylalanine mutants, followed by the analysis of Tau phosphorylation and aggregation. Phosphorylation analysis using the T181 phospho-specific antibody revealed reduced Tau phosphorylation in cells expressing receptor mutants (Fig. 7C and D), with statistically significant effects obtained for the F278A and F278A;F281A mutants (WT: 100% ± 24.6% vs. F278A: 62.6% ± 6.9%, F278A;F281A: 54.3% ± 6.5%). In addition, we found a significantly decreased number of Tau aggregate-positive cells after expression of F281A and F278A;F281A mutants (Fig. 7E; WT: 100% ± 2.7% vs. F281A: 76.1% ± 8.4%; F278A;F281A: 67.6% ± 3.8%). Taken together, these findings highlight the importance of ICL3 (and in particular F278 and F281) for 5-HT7R-induced CDK5 activation resulting in Tau pathology.

### Refinement of the interaction interface model

Based on the above-mentioned experimental results, we refined our initial model using a molecular dynamics simulation. To achieve a refined model structure, we performed 400 ns non-constraint, all-atom MD simulations for *h*5-HT7R/CDK5 and *m*5-HT7R/CDK5 models in an explicit POPC lipid bilayer. In the case of *h*5-HT7R/CDK5, the protein complex quickly re-arranged, and the complex conformation remained stable after 100 ns (protein backbone RMSD ~ 4 Å for 5-HT7R and ~ 2 Å for CDK5). For the *m*5-HT7R/CDK5 complex, the system stabilized after 250 ns (Additional file 14). As expected, most fluctuations occurred in the ICL3 of 5-HT7R and T-loop region of CDK5, as they are the most flexible areas. As a result of the MD simulations, the number of favorable interactions between the proteins was retained, and the interaction surface area was slightly increased (Additional file 15).

**Fig. 8:**
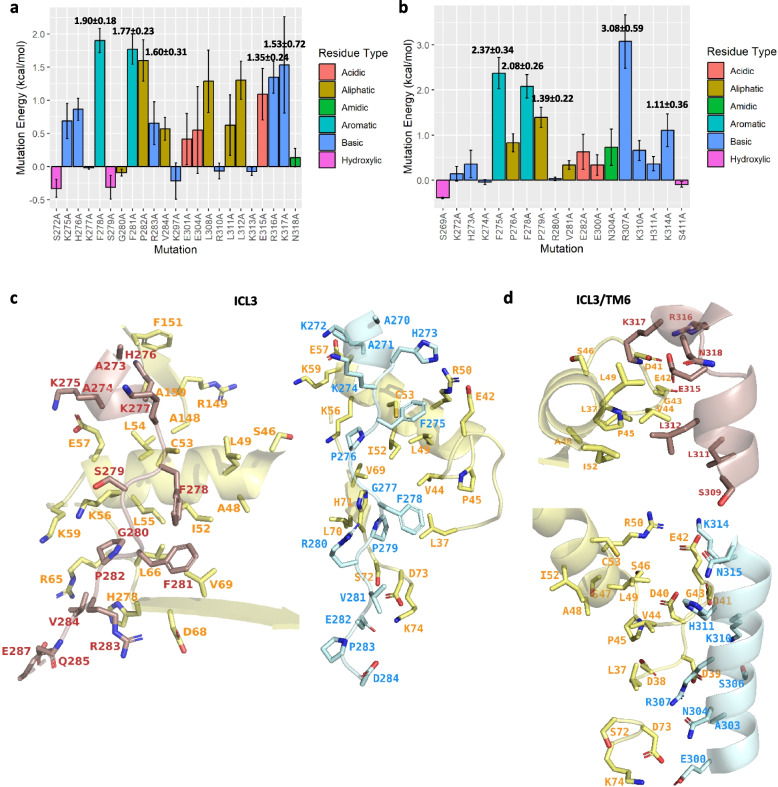
Mutation binding energy calculations in the 5-HT7R/CDK5 models refined by molecular dynamics. **A** PPI binding free energy mutation analysis of the *m*5-HT7R/CDK5 model. Mutation energy values for the top 5 disrupting residues are labeled. **B** PPI binding free energy mutation analysis of the *h*5-HT7R/CDK5 model. Mutation energy values for the top 5 disrupting residues are labeled. **C** 3D view of 5-HT7R residues interacting with CDK5 ɑC-helix hydrophobic surface **D** 3D view of the 5-HT7R residues interacting with CDK5 charged anionic N-lobe surfaces. In structural images, CDK5 residues are shown in yellow, *m*5-HT7R – red, *h*5-HT7R – sky blue

To determine the residues mostly responsible for the protein complex stability, we calculated the energetic effect of alanine substitutions within the 5-HT7R interaction interface in screenshots of the last 10 ns of the MD simulation (Fig. 8A and B). The *h*5-HT7R/CDK5 and *m*5-HT7R/CDK5 complexes were predicted to be stabilized mainly through aliphatic and aromatic residues F278, F281, and P282. As predicted in the initial model, the phenylalanine residues bind preferentially to a hydrophobic surface created by the CDK5 ɑC-helix and N-lobe residues L49, I52, C53, L55 and V69 (Fig. 8C). As a result of the refinement, the 5-HT7R residue H276 moved away from CDK5 E57, losing its charge interaction. For K275A, the binding energy was estimated to be significantly lower than that of phenylalanine residues, suggesting that K275 is responsible only for a weak interaction. Of note, we obtained some differences between human and mouse protein complex models. In the *h*5-HT7R/CDK5, R307 appeared among the top five amino acids responsible for complex stability, which was not the case for the *m*5-HT7R/CDK5 model. Upon closer examination, R307 was localized in the close proximity to CDK5 amino acids D38, D39 and D73 (Fig. 8D). The* m*5-HT7R/CDK5 model proposes some additional hydrophilic interactions involving 5-HT7R residues K317, R316 and E315 and CDK5 residue E42, which is also involved in p25 binding. However, based on the energy analysis, the collective effect of hydrophilic residues on the complex stability is less than that of hydrophobic residues. Therefore, we conclude that the binding interface between 5-HT7R and CDK5 consists mainly of favorable hydrophobic contacts, with F278 and F281 being predominant.

## Discussion

Although multiple studies report important neuronal functions of the 5-HT7R and provide evidence for its involvement in the pathogenesis of several neurological diseases, the underlying molecular mechanisms are still not completely elucidated [59]. We have recently demonstrated that under basal conditions, 5-HT7R physically interacts with CDK5, which results in kinase activation leading to pathological Tau aggregation, impaired LTP, and cognitive deficits in mice [14, 36]. In the present study, we combined molecular biological and biochemical approaches with computational modeling of the 5-HT7R/CDK5 complex to predict and verify residues important for the interaction.

It has been shown that 5-HT7R, unlike most other GPCRs, can be pre-associated with the Gαs subunit even prior to receptor activation [42, 60]. This phenomenon, known as “inverse coupling,” is thought to be responsible for atypically high constitutive 5-HT7R activity. Since CDK5 is also associated with the 5-HT7R under basal conditions, it could be therefore assumed that 5-HT7R, CDK5, and Gs counteract to form the trimeric complex. However, based on the results of our Gαs knock-down experiments, we concluded that Gs protein is not involved in the 5-HT7R/CDK5 complex formation.

**Fig. 9:**
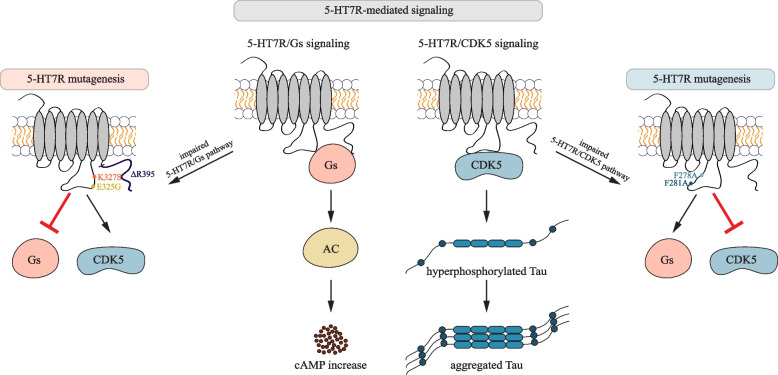
Graphical summary. The 5-HT7R WT stimulates the Gs signaling pathway resulting in increased cellular cAMP concentrations. In addition, 5-HT7R WT activates the Tau kinase CDK5 in G protein-independent manner leading to pathological Tau hyperphosphorylation and subsequent Tau aggregation (middle panel). 5-HT7R mutants that impair the Gs-mediated signaling do not affect the CDK5 signaling (left panel), while 5-HT7R mutants that inhibit the 5-HT7R/CDK5 interaction block the CDK5 signaling without affecting physiological Gs signaling (right panel)

In several previous works, the proximal part of ICL3 as well as the C-terminus of 5-HT7R were identified as receptor domains involved in coupling and/or activation of Gs protein [44, 61]. In particular, 5-HT7R E325G and K327S mutants have been shown to markedly impair ability of receptor to stimulate adenylyl cyclase [44]. Our experiments confirmed the importance of E325, K327, and C-terminus for Gs protein-mediated signaling (Fig. 9). More importantly, our data revealed that receptor domains involved in Gs coupling and Gs protein-dependent signaling neither influenced 5-HT7R/CDK5 interaction nor receptor-mediated Tau hyperphosphorylation and aggregation. Remarkably, we observed that the deletion of the 5-HT7R C-terminus results in an even increased interaction of 5-HT7R with CDK5. Presumably, this large domain might sterically block the access of CDK5 to its docking sites within the receptor. Interestingly, in contrast to the 5-HT7R, another member of the serotonin receptor family, the 5-HT6R, has been shown to constitutively interact with CDK5 via its C-terminal domain [62–64]. In this case, the activation of CDK5 resulted in the phosphorylation of the C-terminal residue S350 of the 5-HT6R, which was necessary for 5-HT6R-elicited neurite outgrowth [63]. Therefore, G protein-independent activation of CDK5 might be a general feature of serotonin receptors, although the interaction interface and functional consequences seem to be receptor-type specific.

To predict 5-HT7R/CDK5 specific interaction interface, we applied AI-based platform ColabFold. In this respect, we first demonstrated on the example of the experimentally determined 5-HT7R/Gs complex that ColabFold can generate 5-HT7R protein complexes with acceptable accuracy. This conclusion was based on the Alpha Fold pTM scores and RMSD-based CAPRI scoring, which quantifies the per-atom position similarity of the computationally generated complex to the experimentally determined one. The AI algorithm correctly predicted the structurally ordered areas of the protein complex and showed the same key interactions, like those known for K327 [48]. Direct comparison of the predicted 5-HT7R/CDK5 protein complex with the published CDK5/p25 crystal structure [49] revealed that the 5-HT7R residues 276–282 could comprise the ICL3 signaling motif responsible for CDK5 activation. Interestingly, although ICL3 represents an intrinsically disordered region in multiple GPCRs, it thought to undergo disorder-to-order transitions to achieve so-called “anchor points” for interactions with specific cytosolic proteins [65, 66]. When activated by p25, the CDK5 PSSALRE helix and N-lobe are rearranged to stabilize the active CDK5 conformation [49, 67]. The close contact of residues F278 and F281 of 5-HT7R to the CDK5 αC helix and N-lobe suggests that these residues strongly contribute to rearrangement and proper positioning of K33 and E51 residues, which are crucial for CDK5 activation. Since the ICL3 sequences of human and mouse 5-HT7R share 98% similarity and 90% identity, we expect that they bind to and activate CDK5 through the same key residues.

Our data also suggests that in addition to CDK5 stimulation via p25-like structure within the ICL3 of 5-HT7R, additional mechanisms may be involved in receptor-mediated CDK5 activation. It has been reported that CDK5 activity can be modulated by its posttranslational modifications, including phosphorylation at Y15 [68, 69] and S159, which might increase CDK5 activity [56–58]. Functionally, S159 in the T-loop of CDK5 is critical for p25 and p35 recognition [49] and might thus also contribute to the selectivity of the CDK5/5-HT7R interaction. In line with this assumption, we demonstrated that phosphorylation at S159 is reduced by phenylalanine mutations suggesting that 5-HT7R might boost CDK5 activation by promoting its phosphorylation at this site. A similar mechanism has been described for the Cables protein, which can form a complex with inactive CDK5, thereby promoting its phosphorylation at Y15, ultimately leading to an increase in total CDK5 activity [70]. Moreover, activated Abl kinase has been shown to interact with CDK5 to potentiate CDK5 kinase activity through Y15 phosphorylation [69]. On the other hand, Kobayashi and coworkers demonstrated that Y15 phosphorylation occurred only on monomeric CDK5, while co-expression of CDK5 activators such as p35/p25 inhibited the phosphorylation [71] suggesting that phosphorylation at Y15 is not an activation mechanism of CDK5. Therefore, future studies are needed to further evaluate the interplay between CDK5 phosphorylation and 5-HT7R-mediated CDK5 activation.

## Conclusions

In the present study, we first demonstrated that receptor domains involved in Gs coupling and Gs protein-dependent signaling are not involved in 5-HT7R interaction with CDK5. Searching for structural determinants of 5-HT7R/CDK5 complex, we predicted and experimentally validated 5-HT7R/CDK5 interaction interface, which includes two phenylalanine residues within the third intracellular loop of the 5-HT7R, F278 and F281 (Fig. 9). Nowadays, it is widely accepted that GPCRs can signal through G protein-independent mechanisms to modulate specific cellular responses [72]. Such biased signaling has become increasingly important as a new pharmacological target [72], but to date the development of biased drugs is focused on arrestin-mediated signaling [73–75]. Here, we demonstrated a biased signaling pathway for the 5-HT7R employing CDK5 as a novel G protein-independent effector. Multiple studies reported that Gs protein-mediated signaling of the 5-HT7R conveys important neuroprotective functions, including regulation of cytoskeleton and gene transcription [39]. This implies that structure-based drug design to development specifically target the CDK5 pathway may be beneficial to treat tauopathies without disrupting physiological Gs protein-mediated cellular responses (Fig. 9).

### Supplementary Information

**Additional file 1.** Primer sequences used to introduce mutations into 5-HT7R.

**Additional file 2.** Representative examples of 5-HT7R/CDK5 models proposed by Colabfold.

**Additional file 3.** Description of prepared systems for molecular dynamics simulations.

**Additional file 4.** Co-expression of 5-HT7R and CDK5.

**Additional file 5.** 5-HT7R co-precipitates with CDK5 independently of Gs protein.

**Additional file 6.** Expression of 5-HT7R mutants affecting Gs protein-mediated signaling.

**Additional file 7.** 5-HT-evoked cAMP responses in neuroblastoma cells expressing 5-HT7R.

**Additional file 8.** Co-expression of CDK5 and 5-HT7R mutants affecting Gs protein-mediated signaling.

**Additional file 9.** Selected initial ColabFold protein complex models.

**Additional file 10.** RMSD comparison of ColabFold model protein complex subunits with experimentally available structures.

**Additional file 11.** List of PDB70 template structures used by the ColabFold algorithm for modeling of individual protein complex chains.

**Additional file 12.** Expression of 5-HT7R mutants affecting CDK5 coupling.

**Additional file 13.** Co-expression of CDK5 and 5-HT7R mutants affecting CDK5 coupling.

**Additional file 14.** Molecular dynamics group atom displacement analysis.

**Additional file 15.** Comparison of protein-protein interface area and interactions at the starting point and during the final 10 ns of MD Simulation.

## Data Availability

The datasets used and/or analyzed during the current study are available from the corresponding author on reasonable request.

## Abbreviations

5-HT: Serotonin
5-HT7R: Serotonin receptor 7
AD: Alzheimer’s disease
ANOVA: One-way analysis of variance
CDK5: Cyclin-dependent kinase 5
co-IP: Co-immunoprecipitation
DMEM: Dulbecco’s modified Eagles medium
For: Forward
FSK: Forskolin
FTD: Frontotemporal dementia
FTDP-17: Frontotemporal dementia with parkinsonism linked to chromosome 17
HA: Hemagglutinin
*h*5-HT7R/CDK5: *Homo sapiens* 5-HT7R/CDK5
IBMX: 3-Isobutyl-1-methylxanthin
ICL3: Intracellular loop 3
IP: Immunoprecipitation
*m*5-HT7R/CDK5: *Mus Musculus* 5-HT7R/CDK5
MD: Molecular dynamics
PPI: Protein-protein interface
Rev: Reverse
Scr: Scramble
SD: Standard deviation
shRNA: Short hairpin RNA
