# Supplementary material for: Structural determinants for activation of the Tau kinase CDK5 by the serotonin receptor 5-HT7R
Source: Cell Commun Signal. 2024 Apr 19;22:233. doi: 10.1186/s12964-024-01612-y (PMC11031989; doi:10.1186/s12964-024-01612-y)
Supplement: Supplementary file 1 — Additional file 1. Primer sequences used to introduce mutations into 5-HT7R. [file 12964_2024_1612_MOESM1_ESM.pdf]

Additional file 1. Primer sequences used to introduce mutations into 5-HT7R

Mutation	Primer
**ΔR395 (HA tag)**	for: GATCGAGGATCCATGTACCCATACGACGTCCCAGACTAC<br>rev: GATCGATCTAGATTACAGGTCCCGGTTGAAGAAGGC
**ΔR395**	for: CTTTTATATATGCCTTCTTCAACCGGGACCTGCGGGATCCACCGGTCGC<br>rev: GCGACCGGTGGATCCCGCAGGTCCCGGTTGAAGAAGGCATATATAAAAG
**E325G**	for: CACGAAAGGAAAAACATTTCCATCTTCAAGAGGGGACAGAAAGCAGCCACTACCTTGGG<br>rev: CCCAAGGTAGTGGCTGCTTTCTGTCCCCTCTTGAAGATGGAAATGTTTTTCCTTTCGTG
**K327S**	for: GAAAGGAAAAACATTTCCATCTTCAAGAGGGAACAGAGCGCAGCCACTACCTTGGGGATC<br>rev: GATCCCCAAGGTAGTGGCTGCGCTCTGTTCCCTCTTGAAGATGGAAATGTTTTTCCTTTC
**E325G/K327S**	for:<br>CACGAAAGGAAAAACATTTCCATCTTCAAGAGGGGACAGAGCGCAGCCACTACCTTGGGGATC<br>rev:<br>GATCCCCAAGGTAGTGGCTGCGCTCTGTCCCCTCTTGAAGATGGAAATGTTTTTCCTTTCGTG
**F278A**	for: GGAAGAGCGCAGCCAAACACAAGGCCTCAGGCTTCCCACGCGTG<br>rev: CACGCGTGGGAAGCCTGAGGCCTTGTGTTTGGCTGCGCTCTTCC
**F281A**	for: GCAGCCAAACACAAGTTCTCAGGCGCCCCACGCGTGCAGCC<br>rev: GGCTGCACGCGTGGGGCGCCTGAGAACTTGTGTTTGGCTGC
**F278A/F281A**	for: GGAAGAGCGCAGCCAAACACAAGGCCTCAGGCGCCCCACGCGTGCAGCC<br>rev: GGCTGCACGCGTGGGGCGCCTGAGGCCTTGTGTTTGGCTGCGCTCTTCC
