# Supplementary material for: Structural determinants for activation of the Tau kinase CDK5 by the serotonin receptor 5-HT7R
Source: Cell Commun Signal. 2024 Apr 19;22:233. doi: 10.1186/s12964-024-01612-y (PMC11031989; doi:10.1186/s12964-024-01612-y)
Supplement: Supplementary file 2 — Additional file 2. Representative examples of 5-HT7R/CDK5 models proposed by Colabfold. [file 12964_2024_1612_MOESM2_ESM.pdf]

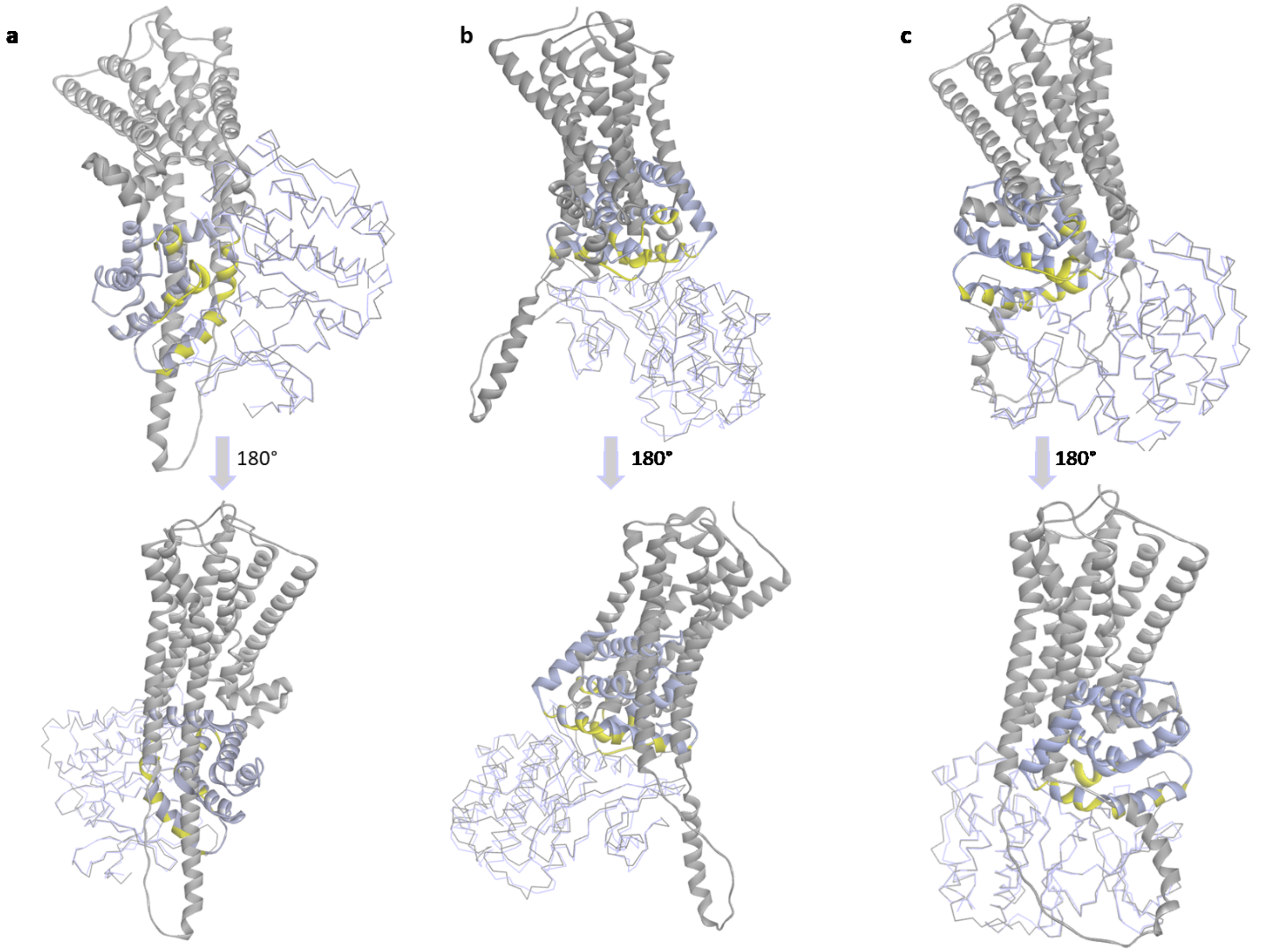

Additional file 2. Representative examples of 5-HT7R/CDK5 models proposed by Colabfold.

Groups 1-3 are shown in **a-c**, respectively. 5-HT7R shown as light gray cartoon, p25 – violet cartoon. Model overlay was based on matching CDK5, which is shown in wire form for clarity with color corresponding to either 5-HT7R or p25. p25 residues which are involved in the CDK5 binding interface are highlighted in yellow.
