# Supplementary material for: Structural determinants for activation of the Tau kinase CDK5 by the serotonin receptor 5-HT7R
Source: Cell Commun Signal. 2024 Apr 19;22:233. doi: 10.1186/s12964-024-01612-y (PMC11031989; doi:10.1186/s12964-024-01612-y)
Supplement: Supplementary file 3 — Additional file 3. Description of prepared systems for molecular dynamics simulations. [file 12964_2024_1612_MOESM3_ESM.pdf]

Additional file 3. Description of prepared systems for molecular dynamics simulations.

System	Model	Cell	POPC Lipid count	Sodium ion count	Chlorine ion count	TIP3P water count	System size, nm
1	*m*5HT7/CDK5	hexagon	400	137	156	50159	13.12* 11.36*15.378*6.56
2	*h*5HT7/CDK5	hexagon	478	154	171	56641	4.25 *12.34 *14.84*7.13
