# Supplementary material for: Structural determinants for activation of the Tau kinase CDK5 by the serotonin receptor 5-HT7R
Source: Cell Commun Signal. 2024 Apr 19;22:233. doi: 10.1186/s12964-024-01612-y (PMC11031989; doi:10.1186/s12964-024-01612-y)
Supplement: Supplementary file 4 — Additional file 4. Co-expression of 5-HT7R and CDK5. [file 12964_2024_1612_MOESM4_ESM.pdf]

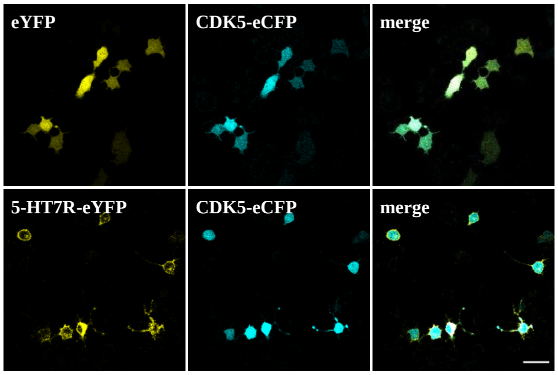

Additional file 4. Co-expression of 5-HT7R and CDK5.

Representative confocal images of N1E-115 cells co-expressing either CDK5-eCFP and eYFP or CDK5-eCFP and 5-HT7R-eYFP. Scale bar: 50 µm.
