# Supplementary material for: Structural determinants for activation of the Tau kinase CDK5 by the serotonin receptor 5-HT7R
Source: Cell Commun Signal. 2024 Apr 19;22:233. doi: 10.1186/s12964-024-01612-y (PMC11031989; doi:10.1186/s12964-024-01612-y)
Supplement: Supplementary file 5 — Additional file 5. 5-HT7R co-precipitates with CDK5 independently of Gs protein. [file 12964_2024_1612_MOESM5_ESM.pdf]

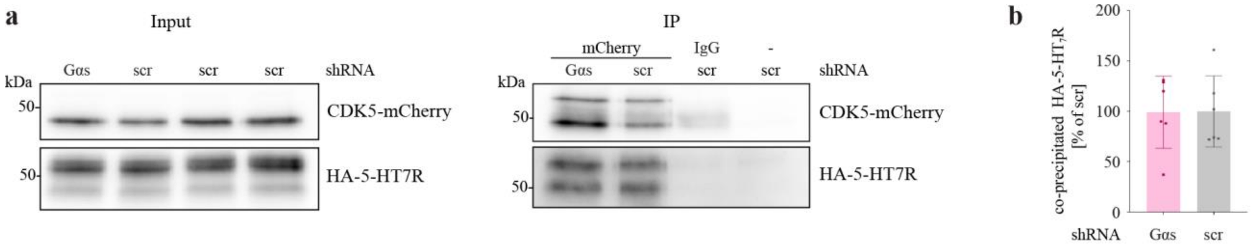

Additional file 5. 5-HT7R co-precipitates with CDK5 independently of Gs protein.

a. Representative co-immunoprecipitation experiment of lysates from neuroblastoma cells and **b.** quantification. Cells co-expressed HA-5-HT7R and CDK5-mCherry (Input). Additionally, $G_{\alpha S}$ protein was knocked down using shRNA. As a control an unspecific sequence (scramble, scr) was employed. Pull-down of recombinant CDK5 was performed with a mCherry antibody (IP). Non-specific IgGs and a sample with no added antibody were used as controls for the pull-down. Data is represented as normalized mean ± SD (N = 6, unpaired student´s t-test, no statistical significance to scr).
