# Supplementary material for: Structural determinants for activation of the Tau kinase CDK5 by the serotonin receptor 5-HT7R
Source: Cell Commun Signal. 2024 Apr 19;22:233. doi: 10.1186/s12964-024-01612-y (PMC11031989; doi:10.1186/s12964-024-01612-y)
Supplement: Supplementary file 6 — Additional file 6. Expression of 5-HT7R mutants affecting Gs protein-mediated signaling. [file 12964_2024_1612_MOESM6_ESM.pdf]

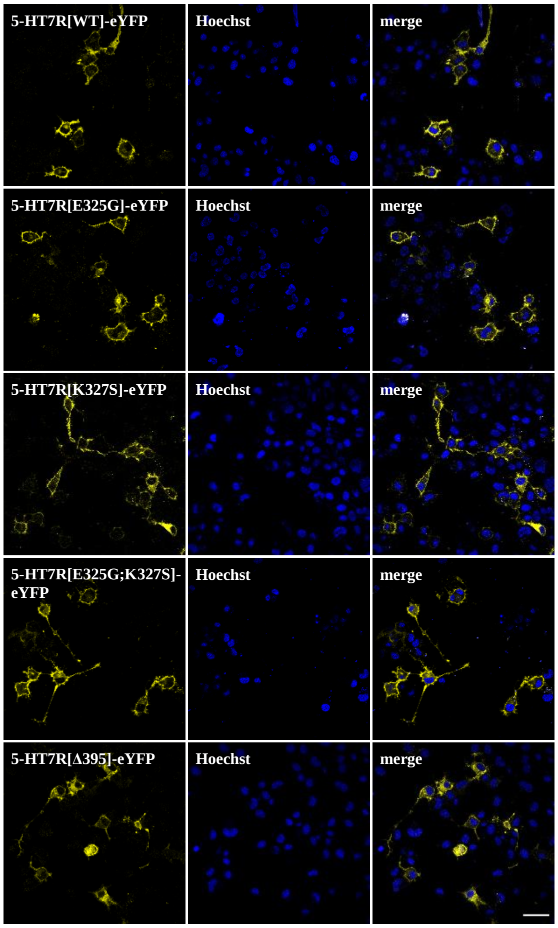


**Additional file 6. Expression of 5-HT7R mutants.**

Representative confocal images showing N1E-115 cells expressing eYFP-tagged 5-HT7R WT or mutants E325G, K327S, E325G;K327S or ΔR395. Nuclei were visualized using Hoechst33342. Scale bar: 50 µm.
