# Supplementary material for: Structural determinants for activation of the Tau kinase CDK5 by the serotonin receptor 5-HT7R
Source: Cell Commun Signal. 2024 Apr 19;22:233. doi: 10.1186/s12964-024-01612-y (PMC11031989; doi:10.1186/s12964-024-01612-y)
Supplement: Supplementary file 7 — Additional file 7. 5-HT-evoked cAMP responses in neuroblastoma cells expressing 5-HT7R. [file 12964_2024_1612_MOESM7_ESM.pdf]

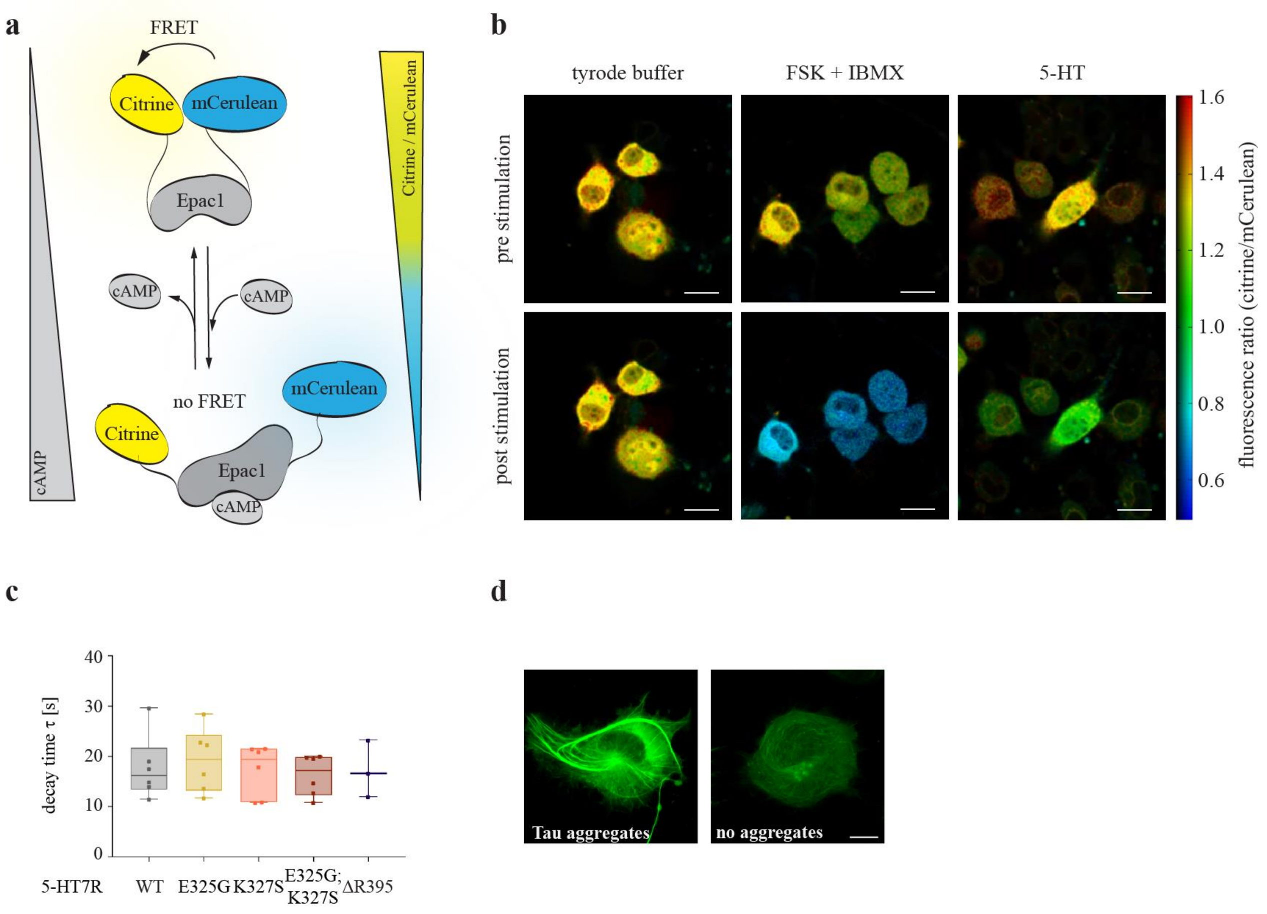

Additional file 7. 5-HT-evoked cAMP responses in neuroblastoma cells expressing 5-HT7R.

a. Scheme of the FRET-based cAMP biosensor. High cAMP levels correlate with low FRET signal between mCerulean (FRET donor) and Citrine (FRET acceptor) calculated by acceptor-to-donor ratio. **b.** Representative N1E-115 cells co-expressing FRET-based cAMP biosensor and 5-HT7R depicted as acceptor-to-donor ratio before and after stimulation with tyrode buffer (negative control), 5 µM forskolin (FSK) and 50 µM 3-Isobutyl-1-methylxanthin (IBMX) (positive control) and 10 µM 5-HT. **c.** Decay time τ of 5-HT-evoked cAMP responses in N1E-115 cells expressing 5-HT7R WT or mutants. Data are presented as box and whiskers min to max (N = 4, Kruskal Wallis test, Dunn's multiple comparisons, no statistical significance to WT). **d.** N1E-115 cell containing aggregates (left) and no aggregates (right).
