# Supplementary material for: Structural determinants for activation of the Tau kinase CDK5 by the serotonin receptor 5-HT7R
Source: Cell Commun Signal. 2024 Apr 19;22:233. doi: 10.1186/s12964-024-01612-y (PMC11031989; doi:10.1186/s12964-024-01612-y)
Supplement: Supplementary file 9 — Additional file 9. Selected initial ColabFold protein complex models. [file 12964_2024_1612_MOESM9_ESM.pdf]

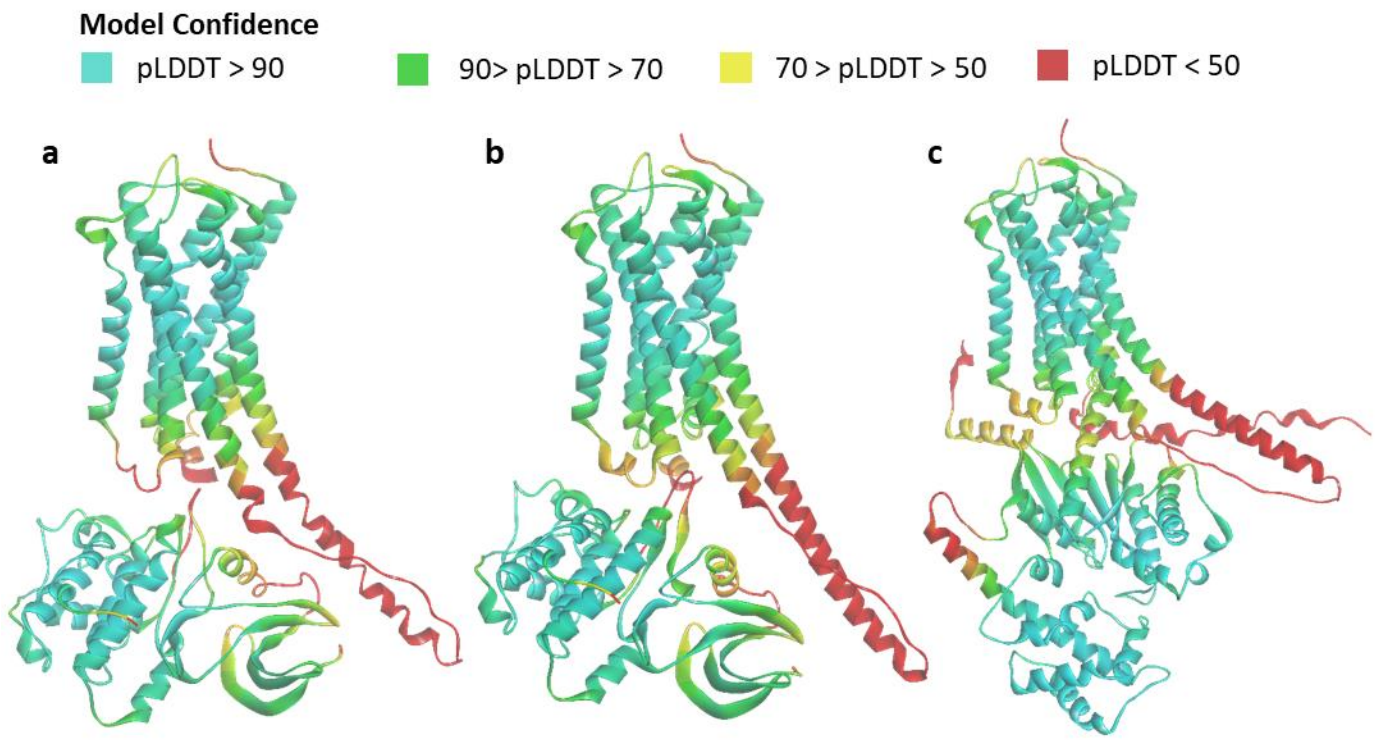

Additional file 9. Selected initial ColabFold protein complex models.

a. *h*5HT7/CDK5, **b.** *m*5HT7/CDK5, **c.** 5HT7/Gαs. Protein colored based on aminoacid pLDDT (confidence) score.
