# Supplementary material for: Structural determinants for activation of the Tau kinase CDK5 by the serotonin receptor 5-HT7R
Source: Cell Commun Signal. 2024 Apr 19;22:233. doi: 10.1186/s12964-024-01612-y (PMC11031989; doi:10.1186/s12964-024-01612-y)
Supplement: Supplementary file 10 — Additional file 10. RMSD comparison of ColabFold model protein complex subunits with experimentally available structures. [file 12964_2024_1612_MOESM10_ESM.pdf]

Additional file 10. RMSD comparison of ColabFold model protein complex subunits with experimentally available structures.

Model	Subunit protein (PBD ID)	RMSD		
		Ca	Main chain	Side chain
*h*5HT7/CDK5	CDK5 (7VDP)	1.980	1.991	2.877
	5HT7R (7XTC)	1.436	1.473	2.450
*m*5HT7/CDK5	CDK5 (7VDP)	1.919	1.913	2.362
	5HT7R (7XTC)	1.444	1.454	2.423
5HT7/Gαs	Gas (7XTC)	3.319	3.218	4.593
	5HT7R (7XTC)	2.343	2.371	3.090
