# Supplementary material for: Structural determinants for activation of the Tau kinase CDK5 by the serotonin receptor 5-HT7R
Source: Cell Commun Signal. 2024 Apr 19;22:233. doi: 10.1186/s12964-024-01612-y (PMC11031989; doi:10.1186/s12964-024-01612-y)
Supplement: Supplementary file 11 — Additional file 11. List of PDB70 template structures used by the ColabFold algorithm for modeling of individual protein complex chains. [file 12964_2024_1612_MOESM11_ESM.pdf]

Additional file 11. List of PDB70 template structures used by the ColabFold algorithm for modeling of individual protein complex chains.*

5HT7R			
**PDB ID**	Protein Name	Orgaism	Ligand
**2R4R**	Beta-2 adrenergic receptor	*Homo sapiens*	None
**4AMJ**	Beta-1 adrenergic receptor	*Meleagris gallopavo*	Carvedilol (agonist)
**4IAQ**	5-Hydroxytryptamine Receptor 1B	*Homo sapiens*	Dihydroergotamine
**4IAR**	5-Hydroxytryptamine Receptor 1B	*Homo sapiens*	Ergotamine
**6H7L**	Beta-1 adrenergic receptor	*Meleagris gallopavo*	Dobutamine (agonist)
**6IBL**	Beta-1 adrenergic receptor	*Meleagris gallopavo*	Formoterol (agonist)
**6KR8**	Beta-2 adrenergic receptor	*Homo sapiens*	None
**7BVQ**	Beta-1 adrenergic receptor	*Homo sapiens*	Carazolol
**7CKW**	D(1A) dopamine receptor	*Homo sapiens*	Fenoldopam
**7CKY**	D(1A) dopamine receptor	*Homo sapiens*	PW0464
**7DH5**	Beta-3 adrenergic receptor	Canis lupus familiaris	Mirabegron
**7E2X**	5-Hydroxytryptamine Receptor 1A	*Homo sapiens*	None
**7E32**	5-Hydroxytryptamine Receptor 1D	*Homo sapiens*	Serotonin
**7E33**	5-Hydroxytryptamine Receptor 1E	*Homo sapiens*	Serotonin
**7EXD**	5-Hydroxytryptamine Receptor 1F	*Homo sapiens*	Lasmiditan
**7EZM**	Cholecystokinin A receptor	*Homo sapiens*	None
**7JOZ**	D(1A) dopamine receptor	*Homo sapiens*	Non-catechol agonist
**7JVP**	D(1A) dopamine receptor	*Homo sapiens*	SKF-83959
**CDK5**			
**1OB3**	PfPK5	*Plasmodium falciparum*	None
**1UA2**	CDK7	*Homo sapiens*	ATP
**1UNL**	CDK5/p25	*Homo sapiens*	R-Roscovitine
**1V0B**	PfPK5 (T198A mutant)	*Plasmodium falciparum*	None
**1V0O**	PfPK5	*Plasmodium falciparum*	Indirubin-5-sulphonate
**3GBZ**	CMGC CDK	*Giardia lamblia ATCC 50803*	None
**3GC0**	CMGC CDK	*Giardia lamblia ATCC 50803*	AMP
**3NIZ**	CDK CGD5_2510	Cryptosporidium parvum	ADP
**3QHR**	pCDK2/CyclinA	*Homo sapiens, Mus musculus*	ADP
**4AU8**	CDK5	*Homo sapiens*	Compound 4a
**4KRC**	CDK PHO85	Saccharomyces cerevisiae S288C	ATP-γ-S

**4KRD**	CDK PHO85/Pcl10	Saccharomyces cerevisiae S288C	None
**4YC3**	CDK1/CyclinB1/CKS2	*Homo sapiens*	None
**5G6V**	CDK16	*Homo sapiens*	Inhibitor
**6GU2**	CDK1/CyclinB/CKS2	*Homo sapiens*	Flavopiridol
**6O9L**	CDK7	*Homo sapiens*	None
**6Q4G**	CDK2	*Homo sapiens*	FragLite37
**6Q4K**	CDK2	*Homo sapiens*	FragLite38
**6XBZ**	CDK7	*Homo sapiens*	AGS
**7B5Q**	CDK7	*Homo sapiens*	ICEC0942

Gas

**PDB ID**	Protein Name	Organism	Ligand
**1CIP**	Guanine nucleotide-binding protein G(i) subunit alpha-1	*Rattus norvegicus*	GNP
**1SHZ**	Guanine nucleotide-binding protein G(i) subunit alpha-1	*Rattus norvegicus*	GDP
**1TAD**	Guanine nucleotide-binding protein G(t) subunit alpha-1	*Bos taurus*	GDP
**1TAG**	Guanine nucleotide-binding protein G(t) subunit alpha-1	*Bos taurus*	GDP
**1TND**	Guanine nucleotide-binding protein G(t) subunit alpha-1	*Bos taurus*	GSP
**1ZCA**	Guanine nucleotide-binding protein subunit alpha-12	*Mus musculus*	GDP
**1ZCB**	Guanine nucleotide-binding protein subunit alpha-13	*Mus musculus*	GDP
**3CX8**	Guanine nucleotide-binding protein subunit alpha-13	*Mus musculus*	GDP
**4EKC**	Guanine nucleotide-binding protein G(q) subunit alpha	*Mus musculus*	GDP
**4EKD**	Guanine nucleotide-binding protein G(q) subunit alpha	*Mus musculus*	GDP
**4N0D**	Guanine nucleotide-binding protein G(i) subunit alpha-1	*Rattus norvegicus*	GNP
**5DO9**	Guanine nucleotide-binding protein G(q) subunit alpha	*Mus musculus*	GDP
**6AU6**	Guanine nucleotide-binding protein G(s) subunit alpha isoforms short	*Homo sapiens*	GDP
**6CMO**	Guanine nucleotide-binding protein G(i) subunit alpha-1	*Homo sapiens*	None
**6NE6**	G-protein alpha subunit Galpha7	*Naegleria fowleri*	None

**7BPH**	Guanine nucleotide-binding protein G(s) subunit alpha isoforms short	*Homo sapiens*	GNP
**7FIG**	Engineered guanine nucleotide-binding protein G(s) subunit alpha	*Bos taurus*	None
**7RKF**	Guanine nucleotide-binding protein subunit alpha-11	*Homo sapiens*	GDP
**7SQ2**	Guanine nucleotide-binding protein G(q) subunit alpha	*Mus musculus*	GDP

*The templates chosen to model the 5-HT7R in *h*5HT7/CDK5, *m*5HT7/CDK5 and 5HT7/Gαs were the same in all 3 individual cases. Same for *h*CDK5 and *m*CDK5 – the templates chosen by ColabFold algorithm were the same.
