# Supplementary material for: Structural determinants for activation of the Tau kinase CDK5 by the serotonin receptor 5-HT7R
Source: Cell Commun Signal. 2024 Apr 19;22:233. doi: 10.1186/s12964-024-01612-y (PMC11031989; doi:10.1186/s12964-024-01612-y)

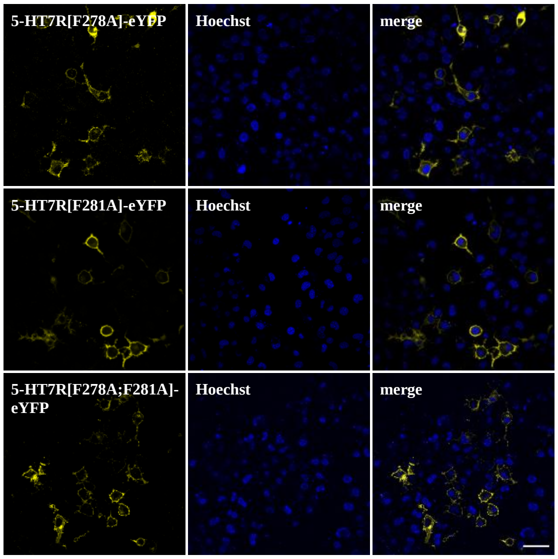

Additional file 12. Expression of 5-HT7R mutants.

Representative confocal images of N1E-115 cells expressing eYFP-tagged 5-HT7R WT or mutants F278A, F281A or F278A;F281A. Nuclei were visualized using Hoechst33342. Scale bar: 50 µm.

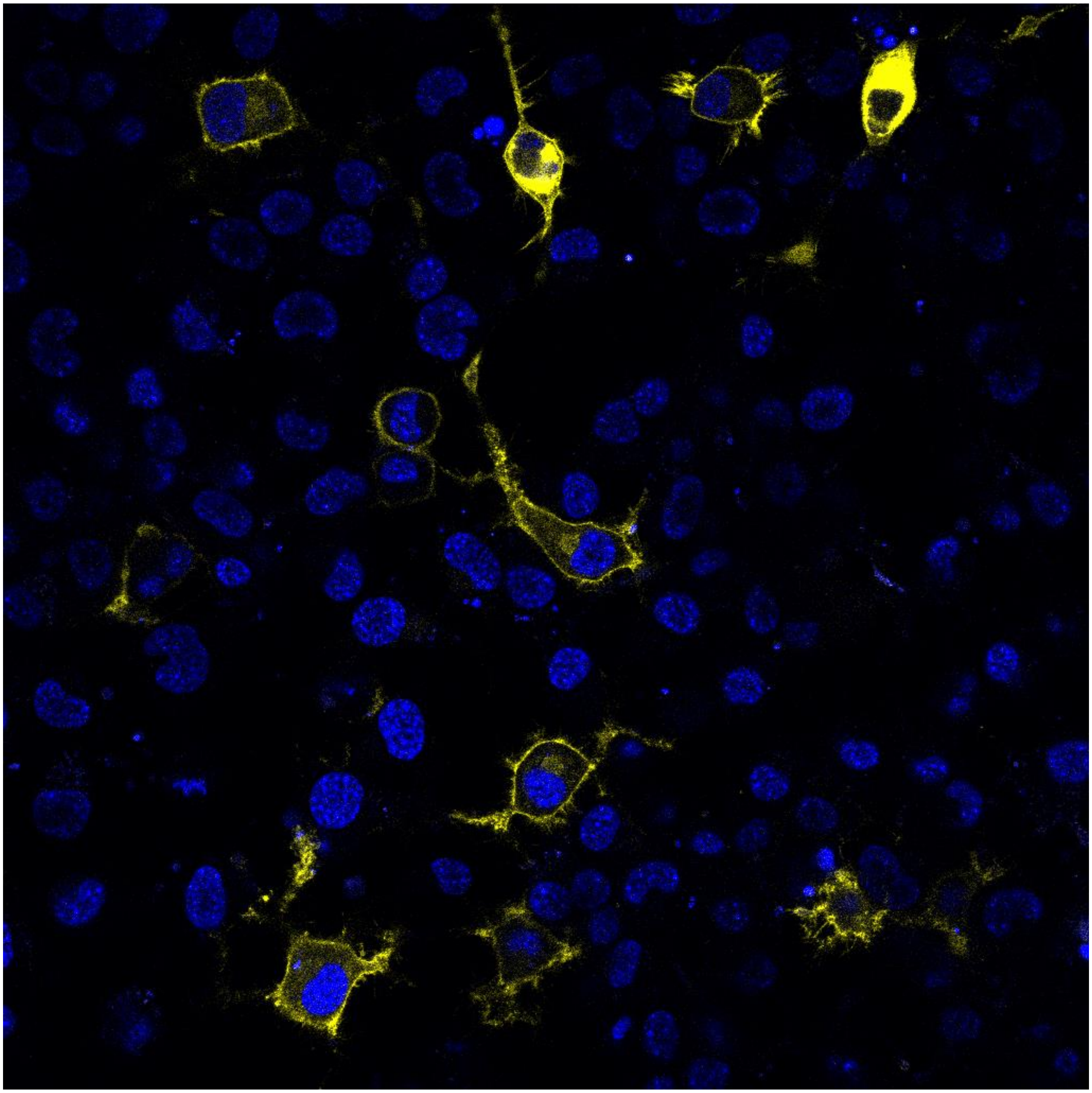

Supplement: Supplementary file 12 — Additional file 12. Expression of 5-HT7R mutants affecting CDK5 coupling. [file 12964_2024_1612_MOESM12_ESM.pdf]
