# Supplementary material for: Structural determinants for activation of the Tau kinase CDK5 by the serotonin receptor 5-HT7R
Source: Cell Commun Signal. 2024 Apr 19;22:233. doi: 10.1186/s12964-024-01612-y (PMC11031989; doi:10.1186/s12964-024-01612-y)
Supplement: Supplementary file 13 — Additional file 13. Co-expression of CDK5 and 5-HT7R mutants affecting CDK5 coupling. [file 12964_2024_1612_MOESM13_ESM.pdf]

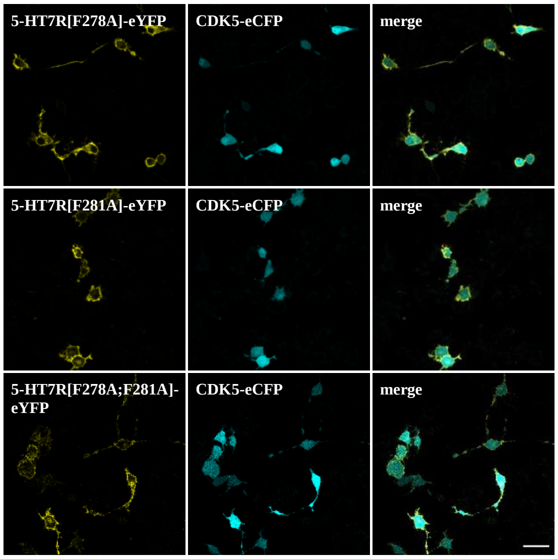

Additional file 13. Co-expression of CDK5 and 5-HT7R mutants.

Representative confocal images of N1E-115 cells co-expressing CDK5-eCFP and eYFP-tagged 5-HT7R mutants F278A, F281A or F278A;F281A. Scale bar: 50 µm.
