# Supplementary material for: Structural determinants for activation of the Tau kinase CDK5 by the serotonin receptor 5-HT7R
Source: Cell Commun Signal. 2024 Apr 19;22:233. doi: 10.1186/s12964-024-01612-y (PMC11031989; doi:10.1186/s12964-024-01612-y)
Supplement: Supplementary file 15 — Additional file 15. Comparison of protein-protein interface area and interactions at the starting point and during the final 10 ns of MD Simulation. [file 12964_2024_1612_MOESM15_ESM.pdf]

Additional file 15. Comparison of protein-protein interface area and interactions at the starting point and during the final 10 ns of MD Simulation.

Model system	MD time, ns	Total Pi Interactions	Total Hydrogen Bonds	Total Salt Bridges	CDK5 Contact Surface Area	CDK5 Polar Contact Surface Area	CDK5 Nonpolar Contact Surface Area	5-HT7R Contact Surface Area	5HT7R Polar Contact Surface Area	5HT7R Nonpolar Contact Surface Area
*m*5HT7/CDK5	0	3	15	2	436.55	228.03	208.52	424.98	154.92	270.06
	390-400	3.18±1.17	14.9±1.44	2.27±0.64	502±59	227±21	275±21	483±59	191±28	291±33
*h*5HT7/CDK5	0	3	9	1	343.53	157.72	185.8	363.26	163.56	199.71
	390-400	2.80±0.63	14.10±2.18	2.70±0.67	483±46	235±27	249±25	489±47	193±30	297±29
